# Assessing the Role of Composition and Size Effects in the Hydrogen Evolution Reaction on Ni_*m*_Pd_*n*–*m*_ Clusters (*n* = 13 and 27)

**DOI:** 10.1021/acsomega.5c05544

**Published:** 2025-09-24

**Authors:** Tiago M. Souza, Henrique A. B. Fonseca, Juarez L. F. Da Silva, Breno R. L. Galvão

**Affiliations:** † 74355 Centro Federal de Educação Tecnológica de Minas Gerais, CEFET-MG, Av. Amazonas 5253, Belo Horizonte, Minas Gerais 30421-169, Brazil; ‡ São Carlos Institute of Chemistry, University of São Paulo, Av. Trabalhador São-Carlense 400, São Carlos, São Paulo 13560-970, Brazil; § School of Physics and Physical Engineering, Qufu Normal University, Qufu, Shandong 273165, China

## Abstract

The low-cost generation of green hydrogen (H_2_) via water electrolysis depends on advances in catalysts for the hydrogen evolution reaction (HER). Although palladium and nickel nanoparticles have demonstrated potential catalytic performance, the exact interplay between particle size and composition in their binary forms remains an unresolved issue. Here, we explore Ni_*m*_Pd_*n*–*m*_ nanoclusters consisting of 13 and 27 atoms to improve our atomistic understanding of the fundamental mechanisms of HER at a nanoscale regime. We used the putative global minimum structure of the bare clusters and employed data mining techniques to generate and select preferential hydrogen adsorption structures. The electronic and geometrical properties are predicted using a cost-effective calculation protocol, based on density functional theory calculations with the PBE functional including van der Waals corrections. The computational hydrogen electrode (CHE) model was employed to calculate Gibbs free energies. We found that atomic H adsorbs on bridge and hollow sites in the 13-atom clusters and only on hollow sites in the 27-atom clusters. The effects of size and composition on the adsorption energies are also calculated, and their relation to the bulk values is presented. Our results on the Gibbs free energy for the HER reaction suggest that, at the subnanometer scale, the most promising catalysts are obtained when earth-abundant nickel clusters are doped with a small percentage of palladium, which has potential application for cost reduction. For instance, Ni_10_Pd_3_ shows a Δ*G* of −0.35 eV, while Ni_13_ and Pd_13_ unary counterparts present −0.51 and −0.47 eV, respectively. An analysis of electron density reveals that within these Ni_*m*_Pd_*n*–*m*_ nanoclusters, a substantial charge transfer from the cluster to the hydrogen atom is correlated with lower values of Δ*G*. This observation implies that charge transfer is a significant factor in the HER.

## Graphical abstract

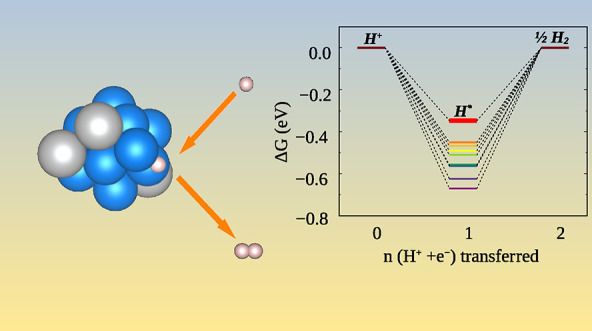

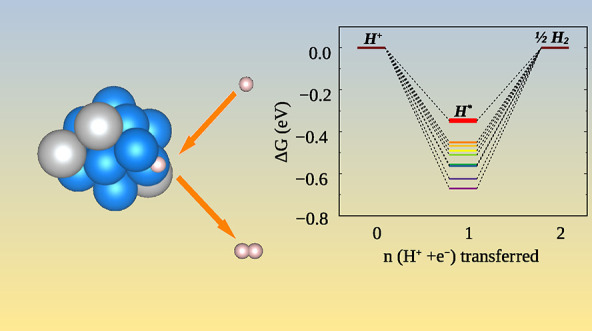

## 1 Introduction

The production of green hydrogen (H_2_) through the electrochemical water splitting process constitutes a substantial alternative for increasing the availability of renewable energy sources.^1,2^ During water electrolysis, the oxygen evolution reaction (OER) occurs at the anode, generating oxygen gas, protons, and electrons. These protons then migrate to the cathode, where they combine with electrons to form a hydrogen gas in the hydrogen evolution reaction (HER).^1^ The reaction process is characterized by three well-established steps,^3,4^ which can be summarized as follows: (i) Initially, proton adsorption occurs at the active site of the catalyst in what is termed the Volmer step. Thereafter, the process may proceed via two distinct pathways: (ii) the Heyrovsky step, where an adsorbed hydrogen reacts with a free proton, and the (iii) Tafel step, where the chemical interaction between two adsorbed hydrogen atoms produces molecular hydrogen.^3,5^

These reaction steps can be expressed by the following chemical equations,

$$H^{+}+e^{-}+*\to H^{*}(\mathrm{Volmer})$$ 1

$$H^{*}+H^{+}+e^{-}\to H_{2}+*(\mathrm{Heyrovsky})$$ 2

$$2H^{*}\to H_{2}(\mathrm{Tafel})$$ 3

where the asterisk * represents an adsorption site. One of the steps above limits the reaction rate and is called the rate-determining step (RDS).^3^ The kinetics of the hydrogen evolution reaction are strongly related to the material used as the electrode, and thus great efforts are devoted to improving the basic electrodes for HER.^3^

Currently, low-cost materials are being evaluated as substitutes for expensive rare metals, which are known for their high catalytic activity in HER.^2,6^ Several transition metal alloys,^2,7^ phosphides,^8^ nitrides,^9^ chalcogenides,^10,11^ and others are being investigated for this reaction.^1,6^ For example, Ni nanoparticles incorporated into a substrate composed of dispersed NiN_*x*_ on porous carbon exhibit high activity in the HER. This catalyst shows an overpotential of 147 mV to achieve a current density of 10 mA cm^–2^ at a low Tafel slope of 114 mV dec^–1^.^6^ Similarly, Pd nanoparticles supported in VS_2_ layers have also shown great potential in HER catalysis in acidic environments. An overpotential of 157 mV at 20 mA cm^–2^ was obtained (measured by linear sweep voltammetry), and the calculations corroborated the experimental findings.^1^

When two or more metal components are mixed, they may show improved catalytic activity due to synergistic effects.^2,12^ This is especially interesting if the catalytic activity of a noble metal can be preserved or enhanced after alloying it with a cheaper earth abundant element. Given the interesting catalytic properties of both the Ni and Pd nanoparticles described above,^1,6^ it would be interesting if the alloy between the two metals at the nanoscale showed similar or superior catalytic activity to the traditional and more expensive catalysts currently employed. It should be highlighted that the properties of NiPd clusters have previously been computationally studied, and the findings suggest that Ni predominantly accumulates at the core of the cluster, whereas most of Pd are placed on the surface.^13–16^ There are several experimental techniques available for the synthesis of such clusters. For example, palladium nanoparticles have been grown in TiO_2_ nanotubes by atomic layer deposition (ALD),^17^ which was subsequently used in ethanol electrooxidation. An alternative approach is the hydrothermal and pyrolysis approach. For example, Ni nanoparticles have been obtained^6^ in this manner using Dicyandiamide and nickel­(II) chloride as precursors.

Catalytic activity is highly influenced by the free energy of hydrogen adsorption (Δ*G*). If the adsorption is too strong, a high overpotential will be required to release it in the form of H_2_. On the other hand, if a positive Δ*G* is observed, an overpotential will be required to adsorb it,^18^ and thus a volcano-type plot is obtained, where the most promising material shows Δ*G* closer to zero.^5,19,20^

Computational simulations of electrocatalytic processes have contributed to our fundamental understanding of several reactions that are important to the energy transition, such as the OER, HER, and the CO_2_ reduction reaction (CO_2_RR).^4,21–25^ For example, it was computationally predicted,^26^ using the computational hydrogen electrode (CHE) model,^21^ that the Mo edge of MoS_2_ had a low Δ*G* for hydrogen adsorption (0.08 eV) and should be active for HER. Later, experiments^27^ confirmed this prediction and showed that the HER activity is indeed linearly correlated with the number of edge sites of MoS_2_ nanoparticles.^25^ Several other computational algorithms have proposed interesting new materials for electrocatalysis.^28–31^

In this investigation, we employed the Ni_*m*_Pd_*n*–*m*_ nanoclusters to explore the effects prompted by variations in composition and cluster size within the HER. Our computational analyzes were performed using density functional theory (DFT), and the structural configurations of the nanoclusters were derived from our prior research study.^16^ We found that hydrogen tends to adsorb at the bridge and hollow sites on the clusters. This preference can be explained by electron transfer, as hydrogen was bonded to atoms that exhibited a negative partial net charge or to atoms with a near-zero charge that were directly bonded to atoms with strongly negative partial charges prior to the adsorption, facilitating electron transfer. We also observed that most clusters exhibited negative Δ*G* values, which may limit their applicability as catalysts. We found the Ni-rich Ni_10_Pd_3_ alloy cluster, as well as the pure unary 27-atom clusters (Ni_27_ and Pd_27_), with Δ*G* values of −0.35 and −0.44 eV, respectively, to be the most promising catalysts among all sizes and compositions assessed. This work provides the theoretical basis for future experimental investigations, providing insight into the catalytic behavior of HER on NiPd nanoclusters.

## 2 Theoretical Approach and Computational Details

### 2.1 Total Energy Calculations

The Vienna Ab initio Simulation Package (VASP)^32^ was used to perform all DFT^33,34^ calculations using the Perdew–Burke–Ernzerhof (PBE)^35^ formulation for the exchange-correlation energy functional, in the spin-polarized framework. The interaction between core and valence electrons was modeled using the projector augmented wave (PAW) method.^36,37^ Furthermore, the semiempirical D3 correction as proposed by Grimme^38^ was incorporated to improve the description of van der Waals interactions.

In order to improve computational efficiency, our DFT-PBE+D3 calculations were partitioned into two distinct stages: low-cost screening optimization and high-cost optimization calculations. Details of the number of calculations for each case are provided in subsequent sections, while here we focus on the computational parameters used in each of them. In the screening phase, we set the cutoff energy of the plane wave at 12.5% lower than the highest recommended value (ENMAX, see Table S1), while the high-cost optimization calculations used a cutoff energy 12.5% above ENMAX. A smearing width of partial occupancies of 0.10 eV was applied in the screening phase and 0.01 eV for the high-cost phase. Geometry optimizations were carried out until the forces in all atoms were below 0.250 eV/Å in the screening phase and 0.050 eV/Å in the high-cost phase. During the initial low-cost screening optimization calculations, in which reduced computational parameters were employed, the cluster atoms remained “frozen” throughout the geometry optimization process. In contrast, in the high-cost stage, these atoms were allowed to undergo relaxation.

To avoid interactions between periodic images, we employed a unit cell with a minimum vacuum of 10 Å for the low-cost screening optimization and 15 Å for the high-cost optimization calculations. Given the large size of the cell, no dispersion in the electronic states is expected within the Brillouin zone (BZ), and only the Γ-point was used for the BZ integration. Additional computational details are reported within the Electronic Supporting Information File.

### 2.2 Computational Hydrogen Electrode Model

To obtain Gibbs free energy changes, the computational hydrogen electrode (CHE) model is employed. In this approach, the reaction $\frac{1}{2}H_{2}⇌H^{+}+e^{-}$ is considered in equilibrium for any temperature and pH.^4^ The free energy (*G*) of each reaction step is calculated as follows:^4,39^

$$G=E_{\mathrm{tot}}+\mathrm{ZPE}+\int C_{p}dT-TS$$ 4

where *E*_tot_ is the total energy obtained from DFT calculations, ZPE represents the zero-point energy, and ∫*C*_p_d*T* represents the enthalpic contribution, where *C*_p_ is the heat capacity at constant pressure and *T* is the temperature. The entropy (*S*) contribution is provided by −*TS*.

Here, we used a temperature of 298.15 K and 1 bar for hydrogen pressure, and the calculations were carried out using the Atomic Simulation Environment package.^40^ This package provides the thermodynamic correction terms from the calculated energies of the vibrational modes (ϵ_*i*_),^4^ as

$$\mathrm{ZPE}=\sum_{i}\frac{1}{2}\epsilon_{i}$$ 5

$$\int C_{p}dT=\sum_{i}\frac{\epsilon_{i}}{e^{\epsilon_{i}/k_{B}T}-1}$$ 6

and

$$S=k_{B}\sum_{i}\frac{\epsilon_{i}}{k_{B}T(e^{\epsilon_{i}/k_{B}T}-1)}-\ln(1-e^{\epsilon_{i}/k_{B}T})$$ 7

where *k*_B_ is the Boltzmann constant. It should be noted that this study does not address kinetic factors such as potential energy barriers.^41^ In the context of water electrolysis reactions, the slow 4-electron oxygen evolution reaction is expected to be the rate-determining step. In contrast, the kinetics of the two-electron transfer mechanism involved in HER are more rapid and should not prevail over the overall kinetics.^42,43^

### 2.3 Adsorbed Structure Configurations

Our previous DFT-PBE investigations analyzed the putative global minima of Ni_*m*_Pd_*n*–*m*_ nanoclusters comprising various sizes (*n* = 13, 27, 41) and seven distinct compositions, wherein the proportion of Ni atoms varied from 12.5, 25.0, 37.5, 50.0, 62.5, 75.0, and 87.5%.^16^ In the current study pertaining to the hydrogen evolution reaction (HER), we concentrated specifically on the 13-atom and 27-atom clusters. This strategic choice enables a comparative analysis of the properties of small subnanometric particles, facilitating a direct evaluation of how the presence or absence of a distinct core affects catalytic behavior. To explore the HER, we begin with the putative global minimum configurations of these nanoclusters. Subsequently, we generated numerous potential adsorption sites for atomic hydrogen on these structures. The objective was to systematically determine the lowest-energy adsorption configurations. This procedure, which is delineated in Figure 1 and elaborated in subsequent sections, acts as a basis to understand the initial stages of hydrogen evolution in these bimetallic nanoclusters.

**Figure 1:**
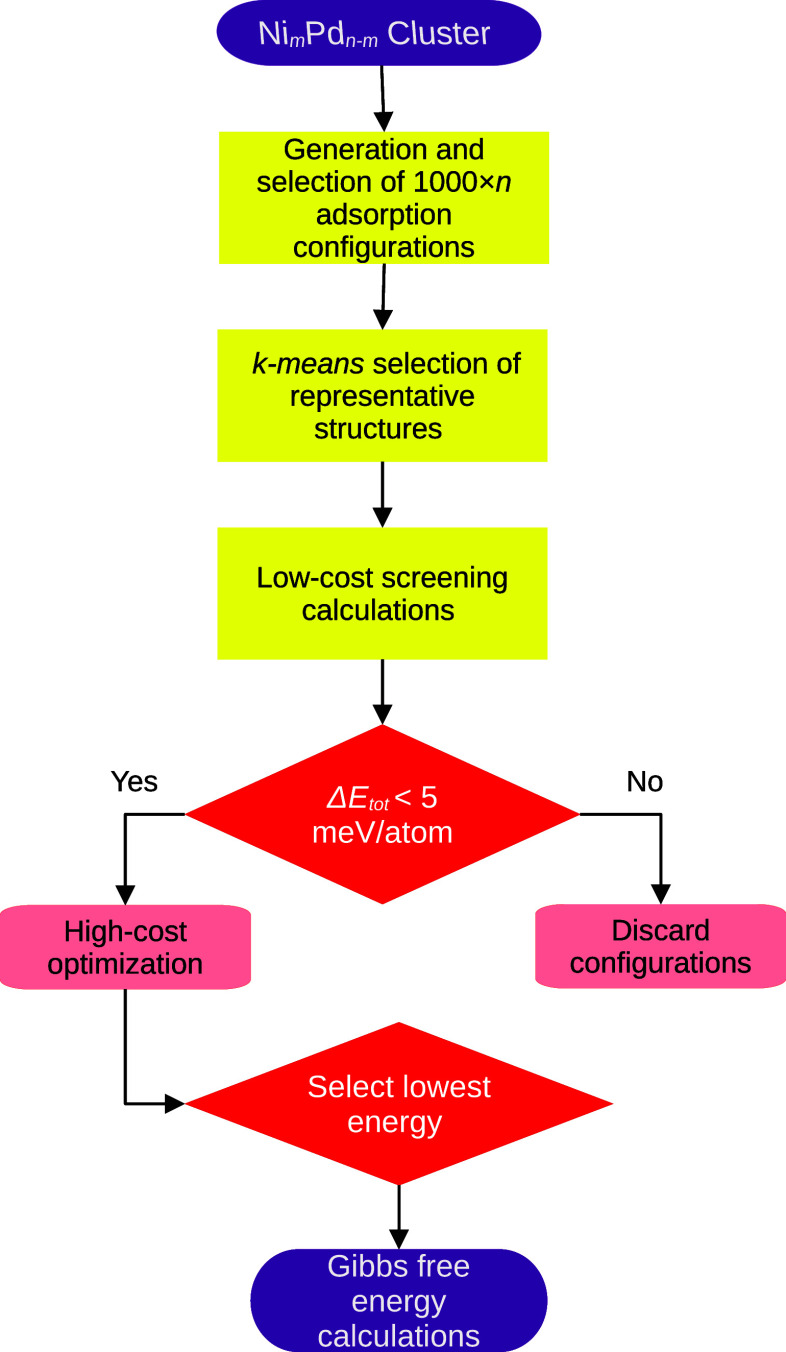
Flowchart describing the protocol to generate and select the adsorbed configurations. Δ*E* is the difference between the total energy of a structure and the lowest energy found.

#### Initial Generation of Adsorbed Configurations

We employ an algorithm described in ref 44 to quickly generate a set of adsorption structures. A large number of positions for the hydrogen atom are generated around the rigid cluster (1 × 10^6^ for clusters with 13 atoms and 2 × 10^6^ for those with 27 atoms). Subsequently, each configuration is converted into a vector with the distances from every atom to the center of gravity of the particle, **R** = (*x*_1_, *x*_2_, ..., *x*_*n*_), where *n* is the number of atoms in the cluster. To remove similar structures, we calculate the normalized distance between two structures *S*(α, β), as follows:^44^

$$S(\alpha ,\beta )=\frac{\sum_{i=1}^{n}(x_{i,\alpha }-x_{i,\beta }{)}^{2}}{\sum_{i=1}^{n}(x_{i,\alpha }^{2}+x_{i,\beta }^{2})}$$ 8

Similar structures are removed if the value of *S*(α, β) falls below a certain threshold, which was selected to achieve the 1000 × *n* configurations. Note that, at this point, the problem of permutation of equivalent atoms does not need to be taken into account, given that all configurations are generated with a rigid cluster.

#### Clustering via *k*-Means Algorithm

A more refined algorithm is used to further reduce the number of adsorbed structures from thousands to a predefined target number (Ad) that has been set to each each Ni_*m*_Pd_*n*–*m*_ nanocluster independently, to allow obtaining all possible nonequivalent sites on that cluster (see the ESI for details). For this purpose, the *k*-means algorithm is a significant tool to cluster large data sets and divide them into new data subsets based on similarities between points. These subsets are referred to as clusters, and their geometric center, termed the centroid, serves as the most representative point for each cluster. As the algorithm proceeds, the centroid’s position for each cluster shifts, enhancing the data distribution within each group. Upon completion, we obtain the most representative *k*-groups from the data, with each centroid acting as the representative structure for its respective group.^45–48^

Within the *k*-means algorithm, the Coulomb matrix (CM) eigenvalues are used to represent each structure.^47,49^ The matrix components *M*_*ij*_ are obtained as follows:

$$M_{ij}=\{\begin{array}{ll} 0.5Z_{i}^{2.4} & \forall \;i=j\\ \frac{Z_{i}Z_{j}}{\left|R_{i}-R_{j}\right|} & \forall \;i\neq j \end{array}$$ 9

Here, *R*_*i*_ are the Cartesian coordinates of the atom *i* and *Z*_*i*_ its nuclear charge. The nuclear repulsion energy between pairs of *i* and *j* is captured by the nondiagonal elements, while the diagonal elements are related to a fit to approximate the potential energy of the isolated atoms.^47,49^ This representation does not change in terms of rotation and transformation of the entire rigid structure.

#### t-Distributed Stochastic Neighbor Embedding

In order to further minimize computational costs and avoid problems caused by high-dimensional data, we use the t-distributed stochastic neighbor embedding (t-SNE), to reduce the dimensions of the CM feature vector to two dimensions, which is then fed to the *k*-means algorithm.^47,50^ The t-SNE method uses the Student Statistic Distribution to determine the similarities between multiple-dimensional point pairs of sample space, and optimize their positions to provide a lower-dimensional representation that reflects the original data.^50^ By doing that, the t-SNE algorithm tends to keep similar points close together in two dimensions, in which the *k*-means algorithm will be applied for selecting structures.^47^

#### Selection of Representative Structures for the High-Cost Optimization Calculations

Subsequent to selecting the most representative Ad structures for each cluster size and composition using the *k*-means algorithm, we perform their local optimization using DFT calculations. A two-step strategy is implemented for this optimization process. Initially, less accurate, low-cost screening optimization calculations are conducted, succeeded by more accurate, high-cost optimization calculations. The purpose of the screening step is to reduce computational expenses by identifying configurations with higher total energies, which are then excluded from further refinement in the high-cost optimization phase. For the selection of configurations for the final step, we opted for those with energy levels that did not exceed 5 meV/atom above the putative global minimum, based on the optimization results of the screening. The screening optimizations resulted in the identification of several symmetrically equivalent local minima, from which only unique structures were selected to minimize computational load at the high-cost optimization level. This was achieved by calculating the root-mean-square deviation of atomic positions (RMSD) between all pairs of structures, employing a superimposing algorithm^51^ (see the ESI for further details).

## 3 Results and Discussion

Given that our strategies include an investigation of potential adsorption configurations through the use of both low-cost screening optimizations and more high-cost optimization refinements, we initially present the results derived at each stage. This allows for an assessment of the volume of computations performed and the variability of the structures examined. Subsequently, we provide an in-depth characterization of the preferred structure, followed by an analysis of the efficacy of the Ni_*m*_Pd_*n*–*m*_ clusters as catalysts in the HER process and a study of the fundamental mechanisms underlying the reaction.

### 3.1 Characterization of the Energy Distribution Profile

Figure 2 presents the relative total energies (Δ*E*_tot_) for the H/Ni_*m*_Pd_*n*–*m*_ systems. These energies are measured with reference to the lowest energy configuration determined for each size and composition; that is, Δ*E*_tot_ = *E*_tot_^*i*^ – *E*_tot_^lowest^. Panels (a) and (c) present the results of the low-cost screening calculations. After this step, we reoptimize the selected configurations with the high-cost calculations, with the results presented in panels (b) and (d) of Figure 2.

**Figure 2:**
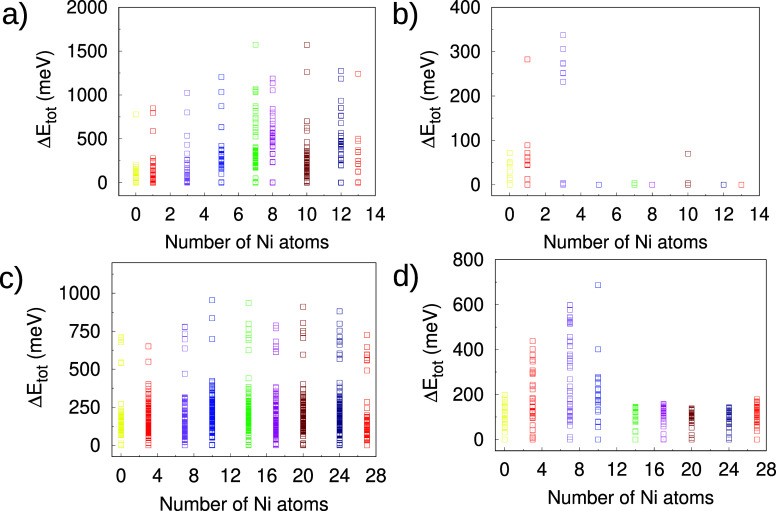
Relative energies for hydrogen adsorption on the Ni_*m*_Pd_*n*–*m*_ clusters: (a) low-cost screening and (b) high-cost optimization calculations for H/Ni_*m*_Pd_13–*m*_; (c) low-cost screening and (d) high-cost optimization calculations for H/Ni_*m*_Pd_27–*m*_.

It can be clearly seen that there are many more points in the low-cost screening optimization results compared to the high-cost counterpart. Recall that the low-cost screening calculations employed a more relaxed tolerance for the geometry convergence, and thus some of the points that are separated at the panels (a) and (c) of Figure 2 may turn out to lead to the same local minimum upon the more rigorous local optimization performed with the high-cost optimization stage, panels (b) and (d). Furthermore, the selection of configurations to be carried out in the final high-cost calculation also contributes to this difference seen in Figure 2. If the putative global minimum is significantly lower in energy than the other local minima, it will lead to the removal of many structures that do not meet the criterion of Δ*E*_tot_ < 5 meV/atom. This is particularly severe for 13-atom clusters that resemble the Ni_13_ unary geometry (truncated triangular bipyramid), while 27-atom clusters tend to suffer less from this effect.

In the case of 13-atom clusters (panels (a) and (b)), preliminary low-cost screenings uncover a broad array of local minima across an energy spectrum reaching approximately 1800 meV. In particular, for intermediate Ni concentrations (ranging from 7 to 10 Ni atoms), a significant density of discrete configurations is detected, emphasizing the intricate adsorption landscape even at such a comparatively small cluster scale. Subsequent high-cost optimization procedures considerably compress the energy distribution, confining it to within 400 meV of the configuration with the lowest energy. Nevertheless, for the majority of compositions, all configurations feature energies in proximity to the local minimum structures.

For 27-atom clusters, low-cost screening effectively reveals a diverse array of local minima, wherein Δ*E*_tot_ values approximate 1000 meV. However, even after intensive optimization processes, significant diversity prevails in the adsorption configurations, manifested as a broad distribution of relative energy values, especially present in compositions with a low or high Ni content. This persistent configurational diversity can be attributed to the increased surface area and the enhanced availability of nonequivalent adsorption sites present in larger clusters, which inherently support a broad spectrum of adsorption geometries. This analysis ultimately highlights the benefit of implementing a dual-step computational approach: an initial low-cost screening phase to thoroughly explore the configurational space, followed by a subsequent high-cost refinement phase to precisely identify thermodynamically significant structures.

### 3.2 Adsorption Site Preferences

Figure 3 presents the lowest energy configurations for hydrogen adsorption on the Ni_*m*_Pd_*n*–*m*_ clusters. It is seen that for all clusters considered, the lowest energy configurations occur exclusively with hydrogen at the bridge or hollow sites. This preference has also been observed in hydrogen adsorption on Cu_55–*n*_ *M*_*n*_ (*n* = Co, Ni, Ru, and Rh), where bridge and 3-fold (hollow) adsorption sites were obtained.^2^ Additionally, we notice hydrogen adsorbs on hollow sites on Ni and Pd unary clusters, as also observed in icosahedral Ni_13_ and the tendency verified in the Pd_13_, Pd_55_ clusters and Pd_55_ surface.^52,53^ Unlike in TiNi_*n*_ (=1–12) where hydrogen tends to adsorb on edge sites of clusters,^54^ we do not observe this tendency for Ni-rich clusters. Although there is a previous study on hydrogen adsorption in NiPd nanoclusters,^55^ no information on the type of sites obtained is available for comparison. Adsorption on bridge sites was only observed in 13-atom clusters, while in the Ni_*m*_Pd_27–*m*_ systems, only hollow sites are preferred. It is also seen that the hydrogen atom has a preference for binding to sites composed of nickel only, whenever those are available at the surface of the cluster, while the converse is not true for the Pd sites.

**Figure 3:**
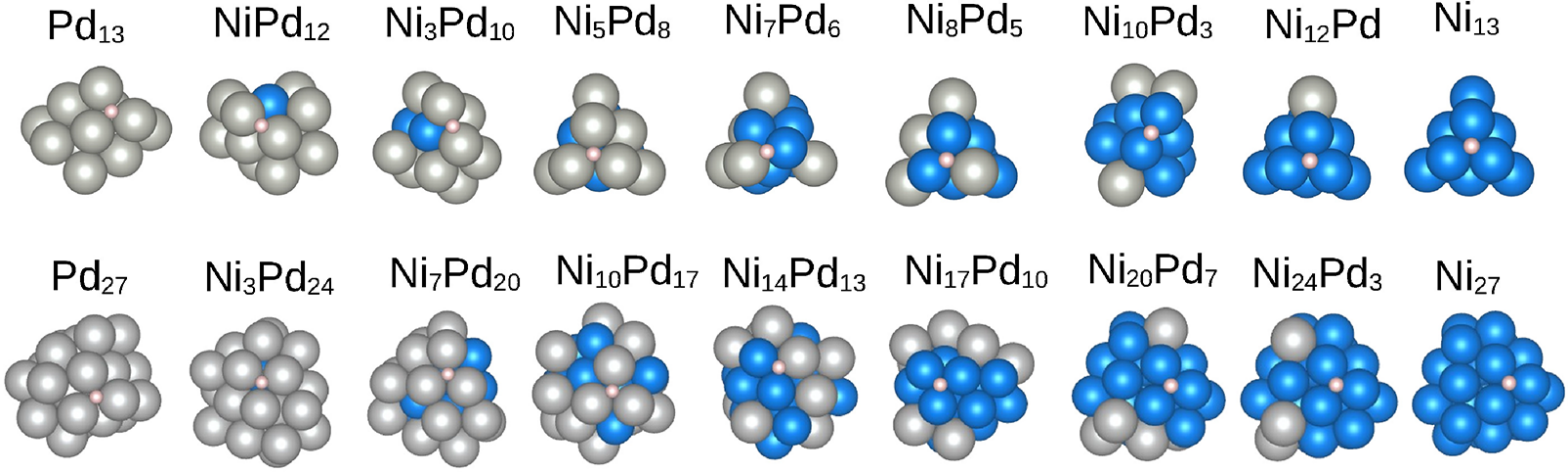
Lowest energy configurations for the adsorption of a hydrogen atom on 13-atoms and 27-atoms clusters.

### 3.3 Geometric Parameters

As shown in Table 1, we found that the lowest distance between H and cluster (*d*_H‑atom_) varies from 1.59 to 1.77 Å for Ni_*m*_Pd_13–*m*_ clusters, while the lowest *d*_H‑atom_ is in the range from 1.67 to 1.81 Å for Ni_*m*_Pd_27–*m*_ clusters. Experimentally, the distance from an adsorbed hydrogen atom to the surface has been reported to be 1.72 Å ± 0.01 Å for 3-fold coordinated sites in Ni(110)^56^ using LEED structural analysis. For Pd(111) surface, the experimental measurement is 1.78 Å ± 0.05 Å.^57,58^ As seen in Table 1, our calculated values are in agreement with the measured ones. This table also provides the effective coordination numbers (ECN, see the ESI) of the metal atoms that compose the adsorption site, which reach up to 7.53 NNN for Ni_*m*_Pd_13–*m*_ and 7.67 NNN for Ni_*m*_Pd_27–*m*_. We could observe that bridge adsorption tends to show low *d*_H‑atom_ (lower than 1.75 Å) and ECN below 7 NNN, while hollow sites tend to show high *d*_H‑atom_ and do not present a clear pattern in terms of ECN. In fact, hydrogen bonds at hollow sites exhibited a wide range of ECN, ranging from 3.99 to 7.67 NNN.

**Table 1:** Adsorption Properties of H Atoms on Ni_*m*_Pd_*n*–*m*_ Clusters (*m* = 13 or 27)^a^

H/Ni_*m*_Pd_13–*m*_					H/Ni_*m*_Pd_27–*m*_				
cluster	site	atom	*d*_H‑atom_	ECN	cluster	site	atom	*d*_H‑atom_	ECN
Pd_13_	hollow	Pd	1.76	3.99	Pd_27_	hollow	Pd	1.86	6.94
		Pd	1.80	4.96			Pd	1.80	6.89
		Pd	1.87	7.53			Pd	1.78	4.94
NiPd_12_	bridge	Pd	1.74	6.72	Ni_3_Pd_24_	hollow	Pd	1.89	7.60
		Pd	1.68	3.93			Pd	1.78	6.74
							Pd	1.81	6.60
Ni_3_Pd_10_	bridge	Pd	1.71	4.91	Ni_7_Pd_20_	hollow	Pd	1.82	7.51
		Pd	1.72	3.71			Pd	1.81	5.71
							Pd	1.83	6.74
Ni_5_Pd_8_	hollow	Pd	1.77	4.89	Ni_10_Pd_17_	hollow	Pd	1.79	5.86
		Pd	1.87	4.87			Pd	1.91	6.35
		Pd	1.80	4.98			Pd	1.81	5.71
Ni_7_Pd_6_	bridge	Ni	1.68	4.80	Ni_14_Pd_13_	hollow	Pd	1.83	7.15
		Pd	1.73	3.81			Ni	1.77	7.44
							Ni	1.69	5.77
Ni_8_Pd_5_	hollow	Pd	1.93	5.00	Ni_17_Pd_10_	hollow	Ni	1.74	6.22
		Ni	1.72	4.82			Ni	1.75	6.54
		Ni	1.72	4.81			Ni	1.67	4.80
Ni_10_Pd_3_	bridge	Ni	1.61	6.80	Ni_20_Pd_7_	hollow	Ni	1.70	5.95
		Ni	1.59	3.84			Ni	1.73	7.27
							Ni	1.73	7.51
Ni_12_Pd	hollow	Ni	1.76	4.98	Ni_24_Pd_3_	hollow	Ni	1.68	5.90
		Ni	1.72	4.84			Ni	1.73	7.54
		Ni	1.74	4.98			Ni	1.74	7.65
Ni_13_	hollow	Ni	1.74	4.98	Ni_27_	hollow	Ni	1.68	5.86
		Ni	1.73	4.98			Ni	1.74	7.67
		Ni	1.74	4.98			Ni	1.74	7.66

a The site type is given, together with the elements that compose the site, their distances to the adsorbed H atom (*d*_H‑atom_ in Å) and their effective coordination numbers (ECN, in Nearest Neighborhood Number, NNN).

### 3.4 Adsorption Energies

The adsorption energy (*E*_ads_) is an important parameter related in the analysis of catalytic activities,^59^ as it encodes the strength of the interaction between the adsorbate and the adsorbent. Here, we assessed the adsorption energy between the cluster and the hydrogen atom for all configurations obtained from high-cost optimization calculations (and so *E*_ads_ is not restricted to the putative global minimum, as usual). The adsorption energy is given by the following equation:

$$E_{\mathrm{ads}}=E_{\mathrm{tot}}^{H/\mathrm{cluster}}-\left(E_{\mathrm{tot}}^{\mathrm{cluster}}+E_{\mathrm{to}t}^{H}\right)$$ 10

where *E*_tot_^H/cluster^ is the total energy of the hydrogen adsorbed configuration in the nanoclusters, while *E*_tot_^cluster^ is the total energy of the isolated nanocluster and *E*_tot_^H^ is the total energy of the H atom.

The results are shown in Figure 4. Starting with the unary cases, we can see that Pd_13_ shows *E*_ads_ varying between −2.9 and −2.8 eV, while the single datum point for Ni_13_ has *E*_ads_ slightly more negative (recall that the reason for fewer data points for 13-atom clusters was highlighted in Section 3.1). This indicates that the hydrogen atom adsorbs more strongly in Ni_13_ than in Pd_13_. This is in agreement with what was obtained in the icosahedral Ni_13_ and Pd_13_, in agreement with previous studies.^55^ We also observed that this trend is reversed in the case of the 27 atoms.

**Figure 4:**
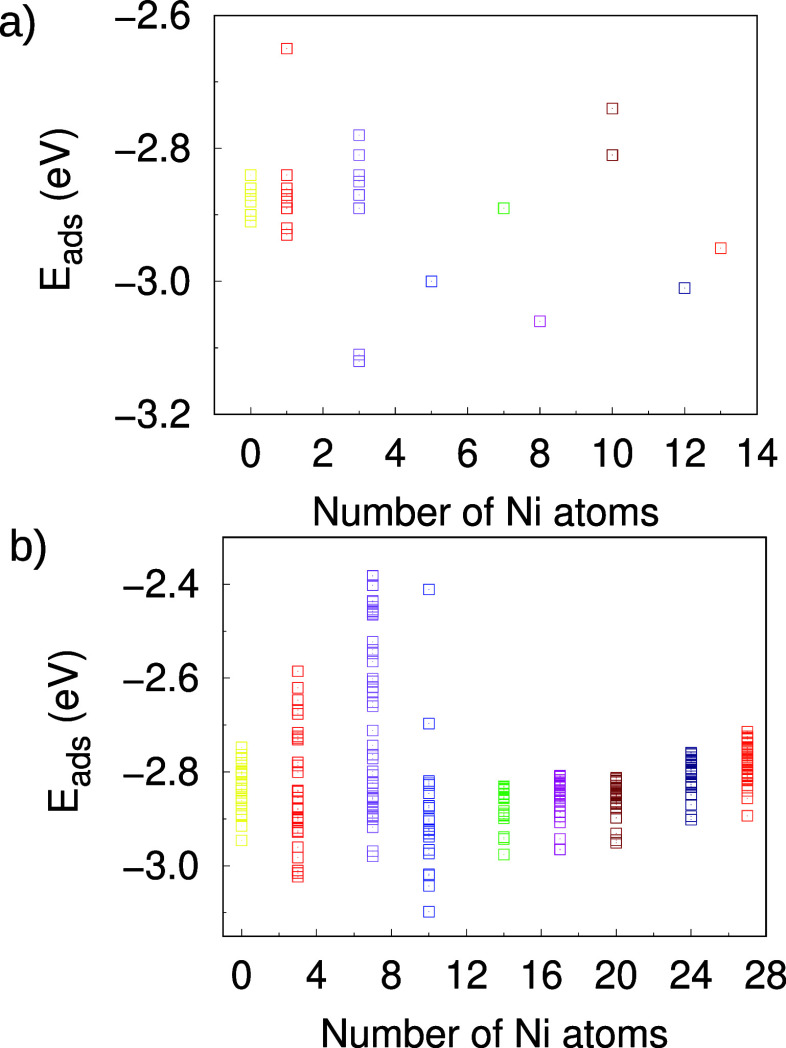
Adsorption energies for the final configurations. Panel (a) shows the Ni_*m*_Pd_13–*m*_ results and panel (b) the Ni_*m*_Pd_27–*m*_ ones.

As illustrated in Figure 4, for both cluster sizes, alloying the Ni and Pd unary clusters leads to an increase in the magnitude of *E*_ads_ (more negative values). The strongest adsorption is observed in the H/Ni_3_Pd_10_ system, in which the hydrogen atom is attached to two Pd atoms in a bridge position, despite having a nickel atom below (see Figure 3). This is in contrast to what has been obtained previously^55^ an an icosahedral NiPd nanocluster with 13 atoms, where alloys were found to bind more weakly. This is possibly due to the lower number of nonequivalent adsorption sites provided by the highly symmetric icosahedral shape as well as the lower number of possibilities for the distribution of Ni and Pd atoms within the particle. Our much larger number of possible adsorption sites allows for the possibility of obtaining not only higher *E*_ads_ than the unary cases but also lower values. The lowest *E*_ads_ values for the H/Ni_*m*_Pd_27–*m*_ system also occurs for the Pd-rich alloys, with a minimum in the H/Ni_10_Pd_17_ case. Here, too, adsorption occurs on palladium atoms, with nickel atoms present in the lower layer.

Overall, the adsorption configurations of H/Ni_*m*_Pd_*n*–*m*_ show *E*_ads_ values ranging from −2.3 to −3.2 eV, as illustrated in Figure 4. Several monometallic and bimetallic NiPd clusters exhibit adsorption energies close to those observed for bulk fcc structures of Ni and Pd, which are −2.71 eV for Ni and −2.68 eV for Pd, respectively.^58^ Furthermore, as the size of the cluster increases, the adsorption energy values for the monometallic clusters become closer to the bulk values. The lowest energy configuration for each composition, shown in Figure 3, was selected for frequency calculations.

### 3.5 Gibbs Free Energies

As remarked in the introduction, if the adsorption is too strong (negative free energy), it will make it difficult to desorb the hydrogen as H_2_,^3^ while a positive free energy of adsorption will hinder the initial (Volmer) step, and for this reason Δ*G* should be as low in magnitude as possible.^60,61^ As shown in Figure 5, Ni_10_Pd_3_ showed the least negative value for Δ*G*, calculated to be around −0.35 eV. This value is substantially lower than the two unary clusters of the same size, indicating that a low percentage of noble palladium atoms in a small NiPd nanoparticle may give rise to synergistic effects that improve the HER performance. This is especially significant given the lowest cost of nickel in comparison to other metals. The Ni_7_Pd_6_ system, with a nearly equal proportion between the two elements, is considered to present a slightly better value than the unary Pd_13_. All other alloys showed a less optimal Δ*G* value than the unary Pd_13_ nanocluster.

**Figure 5:**
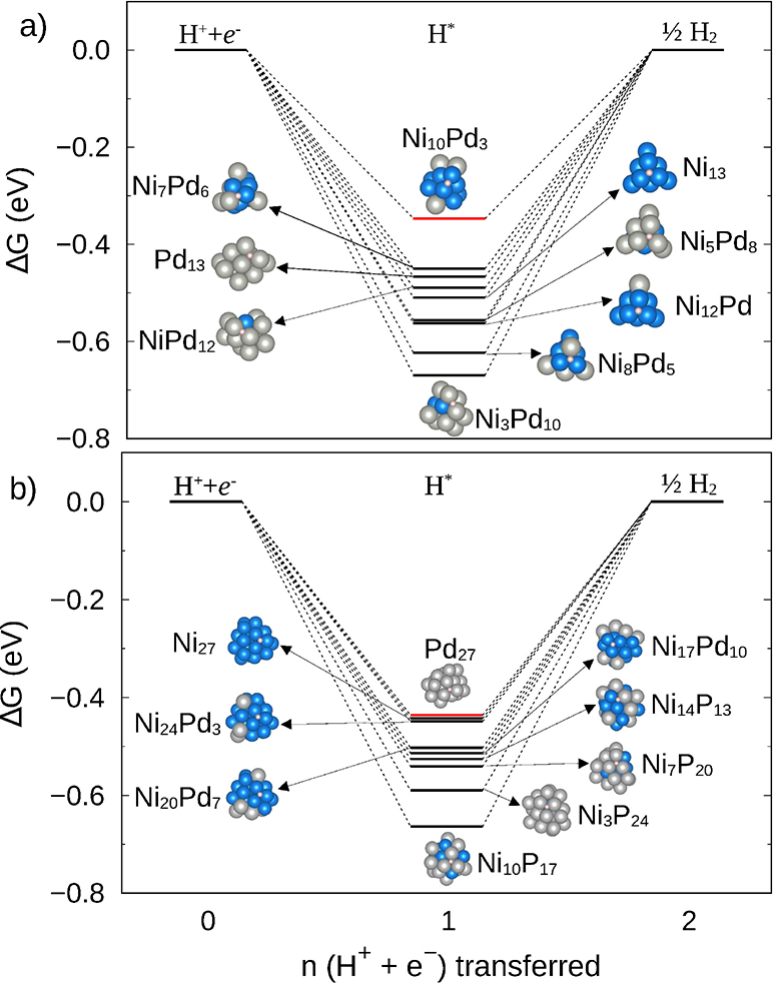
Gibbs free energy diagrams for Ni_*m*_Pd_13–*m*_ clusters in panel (a) and for Ni_*m*_Pd_27–*m*_ in panel (b). The energy of the bare cluster plus an electron proton pair, and the cluster plus molecular hydrogen are represented at the left and right bars, respectively. The structure with highest Δ*G* for each system is marked in red.

For the larger Ni_*m*_Pd_27–*m*_ clusters, however, the unary Pd_27_ and Ni_27_ clusters present the lowest magnitude of Δ*G* (around −0.44 eV), and thus alloying at this cluster size did not show improvements on the catalytic activity. These findings suggest that, at the subnanometer scale, an improved catalytic performance may be obtained by doping nickel with a small percentage of the more expensive palladium atom, but this effect may not be observed for larger clusters. The unique catalytic behavior of very small clusters has been described in the literature.^62^

It is worth mentioning that for the Ni_*m*_Pd_13–*m*_ clusters, as shown in Figure 5, we notice that Ni_13_, Pd_13_, NiPd_12_, and Ni_7_Pd_6_ present Δ*G* between −0.4 and −0.5 eV. These values are close to those observed for the adsorption step in HER on Ru_14_ supported on nitrogen- and sulfur-co-doped carbonaceous shells,^63^ and for hydrogen adsorption on the hollow site of Pd_4_ supported on the VS_2_ surface, where the Δ*G* of hydrogen adsorption value was −0.48 eV.^1^

If we compare only unary clusters, we observe a tendency for Δ*G* to decrease from −0.47 to −0.51 eV when going from Pd_13_ to Ni_13_, while −0.44 eV is obtained for both Pd_27_ and Ni_27_. For bulk surfaces of Ni and Pd, calculations^64^ have found −0.312 and −0.32 eV for Ni(100) and Pd(100), respectively, while for Ni(111) and Pd(111), the values were −0.32 and −0.342 eV, respectively.^64^ Thus, it seems that increasing the size of the unary nanoparticles also decreases the magnitude of Δ*G*, improving the HER process.

### 3.6 Unveiling the Interaction Mechanism

In order to clarify the adsorption preferences, we employ the Density Derived Electrostatic and Chemical (DDEC6) approach^65,66^ to obtain the net atomic charge for each atom in the system. Figures 6 and 7 show the cluster representations and their partial charges distribution before adsorption and after adsorption. For the bare clusters, we can notice a negative net charge accumulation in more coordinated surface atoms, while atoms at the less coordinated edges and vertices tend to present more positive charges. In addition, the palladium atoms tend to have more positive partial charges than the nickel atoms in most of the approached clusters.

**Figure 6:**
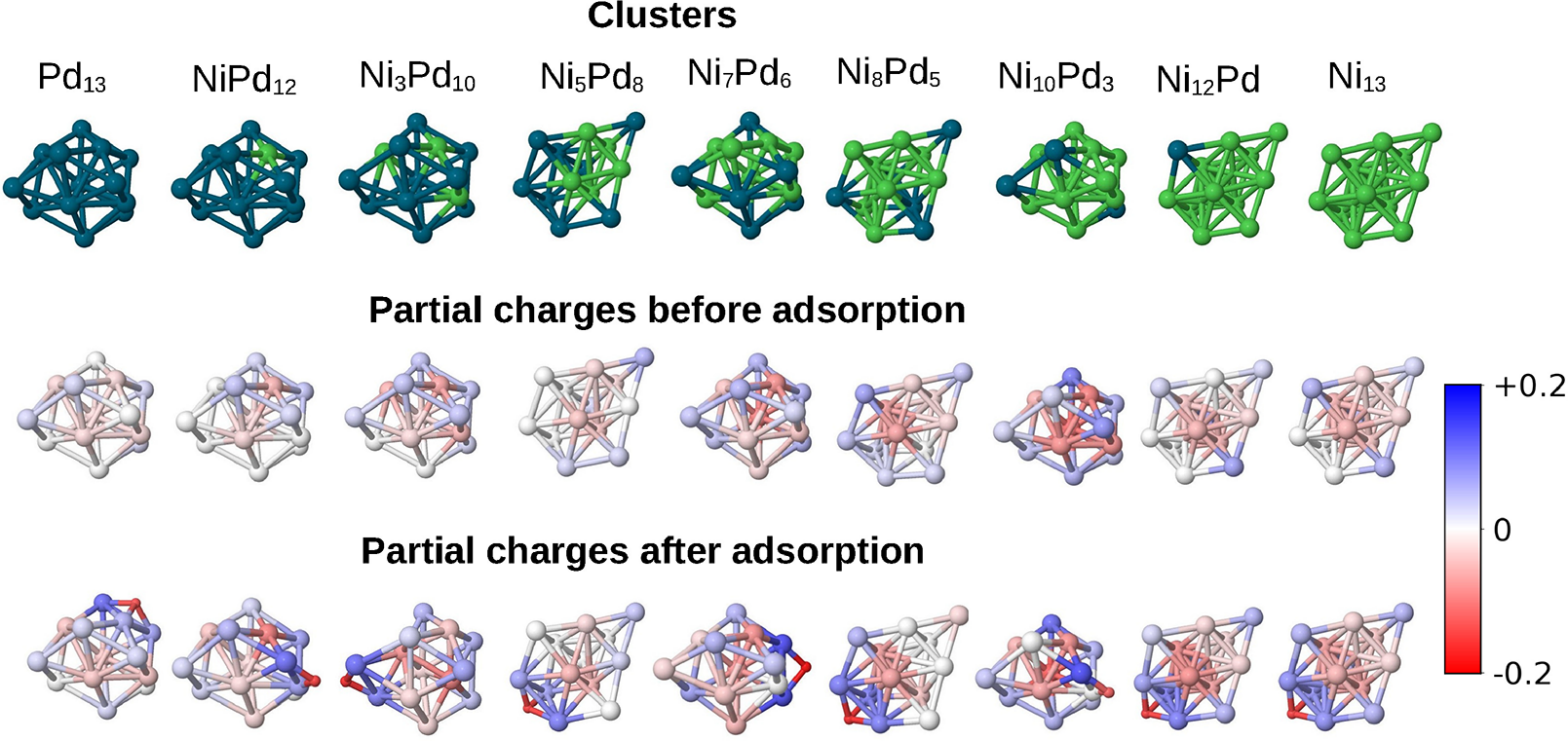
DDEC6 net atomic charge analysis. The top row presents the Ni_*m*_Pd_13–*m*_ clusters for reference (with Ni in green and Pd in dark blue). The middle and lower rows display the partial charges on all atoms before and after adsorption, respectively. Atoms with more negative net charges are represented in darker red, and those more positive are in blue.

**Figure 7:**
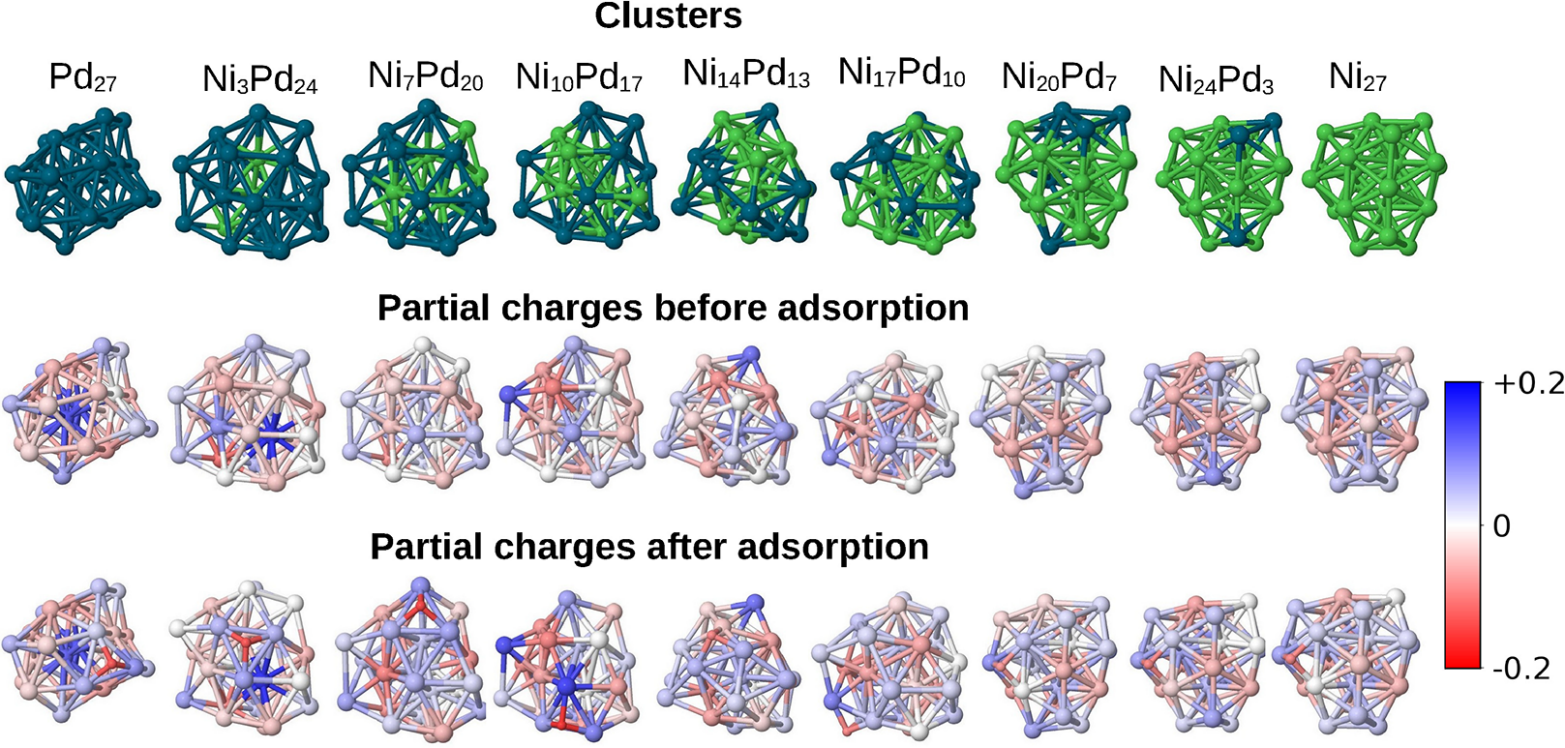
DDEC6 net atomic charge analysis. The top row presents the Ni_*m*_Pd_27–*m*_ clusters for reference (with Ni in green and Pd in dark blue). The middle and lower rows display the partial charges on all atoms before and after adsorption, respectively. Atoms with more negative net charges are represented in darker red, and those more positive are in blue.

Regarding the adsorbed configurations shown in Figures 6 and 7, we can clearly see that hydrogen presents a significant partial negative charge in all cases, indicating electron transfer from the cluster to the hydrogen atom upon adsorption. Concurrently, the site atoms and the atoms directly bonded to them become significantly more positive. Interestingly, the hydrogen atom does not necessarily adsorb on sites that were markedly negative on the bare clusters prior to adsorption, which is indicative of the fact that this process can be better understood in terms of a covalent bond formation. In most 13-atom cases, it adsorbs on atoms with a slightly positive or neutral partial charge in the bare cluster, but these atoms are directly bonded to more negative atoms. The only exception, in which the hydrogen atom adsorbs to a bridge site that had a partially negative atom in the bare cluster, is the case Ni_10_Pd_3_, which was the only alloy that presented a value of Δ*G* that is more promising than the unary nanoclusters.

Figure 8 shows the correlation between electron transfer to the hydrogen atom and the Gibbs free energy associated with HER. First, we notice that the smaller 13-atom clusters present more effective electron transfer than the 27-atom ones. At this lower cluster size, the structure where hydrogen presented the lowest charge transfer was Ni_10_Pd_3_, with −0.106 *e*, which also had a higher Δ*G* than other Ni_*m*_Pd_13–*m*_ systems. Moreover, a larger transfer of electrons from the cluster to the H atom, such as Ni_8_Pd_5_ and Ni_3_Pd_10_ (*Q* = −0.143 and *Q* = −0.141 *e*), are associated with lower Δ*G*. With the exception of Ni_7_Pd_6_, we can see a clear correlation between a larger electron transfer to the adsorbate and a decrease in Δ*G* (stronger adsorption). This behavior is also noticed for hydrogen adsorption on Ni_*m*_Pd_27–*m*_ alloys, where Ni_7_Pd_20_, Ni_3_Pd_24_ and Ni_10_Pd_17_ alloys (which present −0.129, −0.116, and – 0.140 *e* respectively), present low Δ*G*. The unary Ni_27_ cluster also follows this trend, with low charge transfer and weak interaction. Conversely, Pd_27_ shows the same Δ*G* as Ni_27_ (−0.44 eV) but show a more pronounced charge transfer of *Q* = −0.116*e*.

**Figure 8:**
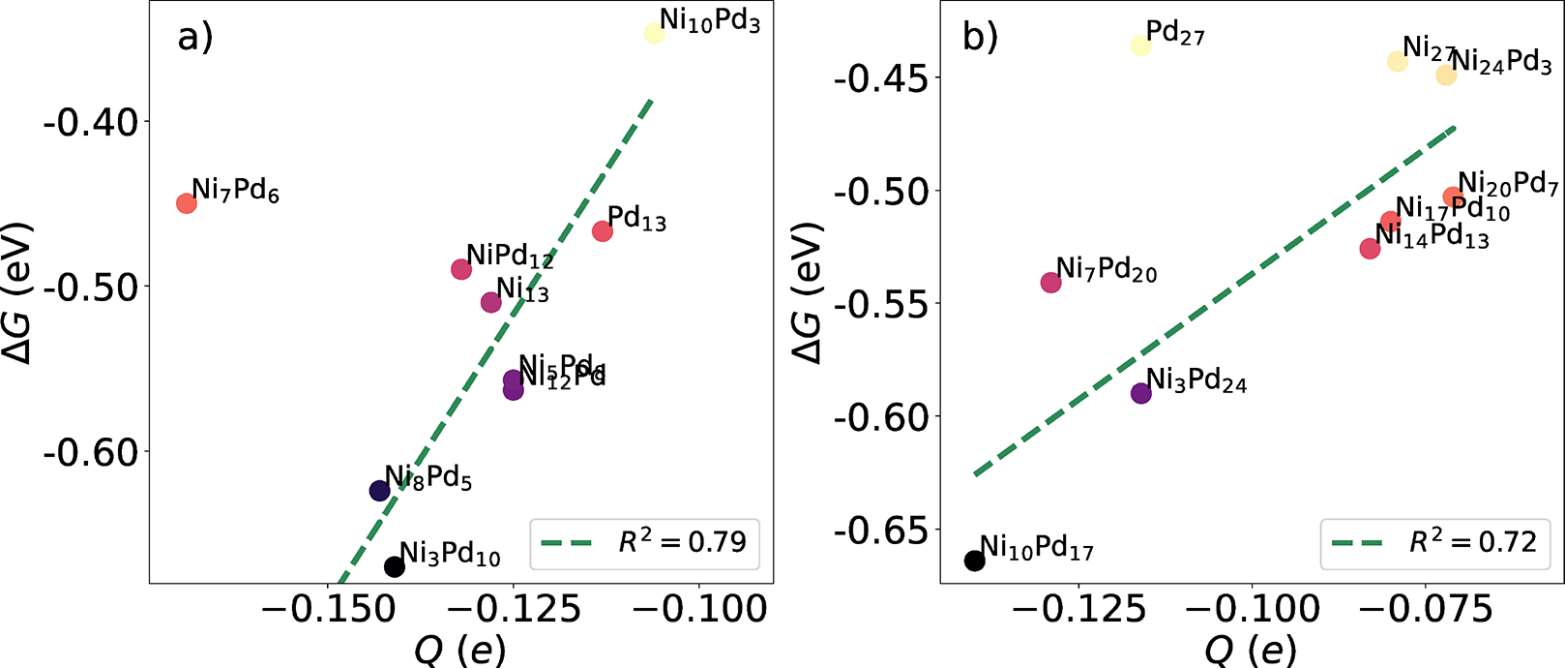
Relation between Δ*G* and effective DDEC charges on the hydrogen atom after adsorption for Ni_*m*_Pd_13–*m*_ (left panel) and for Ni_*m*_Pd_27–*m*_ (right panel). The trending lines and *R* ^2^ values were obtained disregarding the outliers.

## 4 Conclusions

In this work, we conducted a DFT-PBE+D3 investigation on nine compositions in 13- and 27-atom nanoclusters (e.g., 0.0, 12.5, 25.0, 37.5, 50.0, 62.5, 75.0, 87.5, and 100.0% of Ni) to evaluate their performance for the HER. Thus, the selected nanoclusters allowed us to evaluate the effects of size and composition, which is important to optimize the HER performance.

From our calculations and analyses, we found that the adsorption of H in the 13-atom NiPd nanoclusters occurs only in the bridge and hollow sites, while for the 27-atom nanoclusters, the H atom adsorbs exclusively in hollow sites, that is, size effects play a crucial role in the preference of the adsorption site. The reaction free energy varies between −2.3 and −3.2 eV for all configurations, with several NiPd clusters assuming values between −2.68 and −2.71 eV, close to the fcc Ni surface and bulk Pd surfaces, respectively. For Ni_*m*_Pd_13–*m*_ clusters, we noted that the Ni-rich Ni_10_Pd_3_ cluster presented the lowest absolute value of Δ*G*. This points to better catalytic activity compared to both unary clusters of the same size. This, in turn, indicates that at this size scale, earth-abundant nickel can be used with a small percentage of the rare palladium, offering interesting possibilities.

To understand the behavior of Δ*G*, we performed DDEC6 charge calculations. On the basis of the charge distribution, we observed that there is clear electron transfer to the hydrogen atom upon adsorption. In most clusters, hydrogen tends to bind to sites with negative atoms in the vicinity, which likely plays a role in adsorption by improving charge transfer from the cluster to the atomic H. Furthermore, we observed that there is a clear correlation between the magnitude of charge transfer and Δ*G* for HER. In future studies, we plan to investigate the influence of ligands on HER,^67–69^ as they could potentially play a significant role in adsorption, particularly affecting hydrogen adsorption sites.

## Supplementary Material



## Data Availability

All DFT calculations were done using the Vienna Ab initio Simulation package, which can be used under a nonfree academic license. Additional details can be obtained from the link, https://www.vasp.at. The codes used for the data mining tools are available at github.com/lucasbpena/Cluster_k-means.git. Furthermore, additional details are provided within the electronic Supporting Information, while additional crude data can be obtained directly with the authors upon request.

## Notes

Data used for the figures, as well as complementary analyses and additional technical details (PDF)

## Abbreviations

DFT: density functional theory
PBE: Perdew–Burke–Ernzerhof
VASP: Vienna Ab initio Simulation Package
PGMCs: putative global minimum configuration
OER: oxygen evolution reaction
HER: hydrogen evolution reaction
t-SNE: t-distributed stochastic neighbor embedding
CM: Coulomb matrix
