# Supplementary material for: Assessing the Role of Composition and Size Effects in the Hydrogen Evolution Reaction on Ni m Pd n–m Clusters (n = 13 and 27)
Source: ACS Omega. 2025 Sep 24;10(39):45471–81. doi: 10.1021/acsomega.5c05544 (PMC12508918; doi:10.1021/acsomega.5c05544)
Supplement: Supplementary file 1 [file ao5c05544_si_001.pdf]

Electronic Supporting Information: Assessing the Role of Composition and Size Effects in the Hydrogen Evolution Reaction on Ni_mPd_{n-m} Clusters ($n = 13$ and 27)

Tiago M. Souza,[†] Henrique A. B. Fonseca,[‡] Juarez L. F. Da Silva,[‡] and Breno R. L. Galvão[*,†,¶]

[†]*Centro Federal de Educação Tecnológica de Minas Gerais, CEFET-MG, Av. Amazonas* 5253, 30421-169*, Belo Horizonte, Minas Gerais, Brazil*

[‡]*São Carlos Institute of Chemistry, University of São Paulo, Av. Trabalhador São-Carlense 400,* 13560-970*, São Carlos, SP, Brazil*

[¶]*School of Physics and Physical Engineering, Qufu Normal University, Qufu, Shandong 273165, China*

E-mail: brenogalvao@gmail.com

Contents

S-1 Introduction **S-3**

S-2 Additional Details on the Theoretical Approach **S-3**

S-2.1 Selected PAW Projectors S-3

S-2.2 Calculation Parameters S-4

S-2.3 Adsorption and Relative Total Energies S-4

S-2.4 Structural Similarity Analysis via Root Mean Square Deviation Algorithm . S-4
S-2.5 Effective Coordination Concept S-5
S-2.6 Number of Adsorption Sites and Generation of Adsorption Structures S-6

S-3 Additional Data and Complementary Analysis S-7
S-3.1 Selection of Representative Structures for the High-Cost Optimization Calculations S-7
S-3.2 13-atoms Unary and Binary NiPd Clusters S-8
S-3.2.1 Low-Cost Screening Optimization Calculations S-8
S-3.2.2 High-Cost Optimization Calculations S-9
S-3.2.3 Structural Parameters and Free Energy S-10
S-3.3 27-atoms Unary and Binary NiPd Clusters S-13
S-3.3.1 Low-Cost Screening Optimization Calculations S-13
S-3.3.2 High-Cost Optimization Calculations S-14
S-3.3.3 Structural Parameters and Free Energy S-15

S-4 Tables With Cartesian Coordinates and Net Charge Distribution of Non-Adsorbed Configurations S-19
S-4.1 13-atoms Unary and Binary NiPd Clusters S-20
S-4.2 27-atoms Unary and Binary NiPd Clusters S-25

S-5 Tables With Cartesian Coordinates and Net Charge Distribution of Adsorbed Configurations S-34
S-5.1 13-atoms Unary and Binary NiPd Clusters S-34
S-5.2 27-atoms Unary and Binary NiPd Clusters S-39

References S-48

S-1 Introduction

In this document, we present additional technical details, complementary results, and analyses that support the discussion of the main manuscript. Specifically, we include details on the density functional theory (DFT) methodology, a description of the selection and generation of adsorbed configurations, calculation results at the screening level, Spearman correlation analysis, and additional structural and electronic data.

S-2 Additional Details on the Theoretical Approach

S-2.1 Selected PAW Projectors

We used the Vienna *Ab initio* Simulation Package (VASP)[1] to perform Density Functional Theory (DFT)[2,3] calculations using the Perdew-Burke-Ernzerhof (PBE)[4] functional. The Projector Augmented Wave (PAW) method[5,6] was used to describe the interaction between the core and the valence electrons, with details of the projectors given in Table S-1. The semi-empirical D3 correction proposed by Grimme[7] was included.

Table S-1: Technical details of the PAW-PBE projectors selected for this study. Recommended cutoff energy for the plane-wave basis set, `ENMAX` (eV), number of valence electrons, `ZVAL`, and valence electronic configuration

Element	PAW projector	`ENMAX`	`ZVAL`	Valence Config.
H	H_GW 21Apr2008	300.000	1	$1s^1$
Pd	Pd_sv_GW 05Dec2013	356.093	18	$[Ni]4s^2 4p^6 4d^{10}$
Ni	Ni_sv_GW 05Dec2013	389.645	18	$[Ne]3s^2 3p^6 3d^{10}$

S-2.2 Calculation Parameters

Table S-2: Parameters for the DFT calculations for the low-cost screening optimization step, and for the high-cost optimization ones.

	Low-cost	High-cost
Cutoff energy (eV)	341	438
Smearing width of partial occupancies (eV)	0.100	0.010
Geometry optimizations convergence criteria (eV/Å)	0.25	0.05
SCF convergence criteria (eV)	10^{-4}	10^{-5}
Vacuum thickness in unit cell (Å)	10	15

S-2.3 Adsorption and Relative Total Energies

To compare the stability between different configurations with the same cluster size and composition, we calculate relative total energies as:

$$\Delta E_{tot} = E_{tot}^{x} - E_{tot}^{low} , \quad \text{(S1)}$$

where E_{tot}^{low} is the total energy of the lowest energy configuration and E_{tot}^{x} is the total energy of the compared configuration.

The adsorption energy between the cluster and the hydrogen atom was calculated as:

$$E_{ads} = E_{tot}^{\mathrm{H}/cluster} - \left(E_{tot}^{cluster} + E_{tot}^{\mathrm{H}}\right) , \quad \text{(S2)}$$

where $E_{tot}^{\mathrm{H}/cluster}$ is the total energy of the adsorbed configuration, $E_{tot}^{cluster}$ is the total energy of the isolated cluster and E_{tot}^{H} is the total energy of an isolated H atom. We use the E_{ads} variable not only for the putative global minimum configuration of the H atom on each cluster, but for all the other local minima as well.

S-2.4 Structural Similarity Analysis via Root Mean Square Deviation Algorithm

The root mean square deviation of the atomic positions was used to automatically determine if two given configurations are equivalent. For two structures, *A* and *B*, with *n* atoms, the *RMSD* is

calculated as:

$$RMSD = \sqrt{\sum_{i=1}^{n} \| r_A^i - r_B^i \|^2}\,, \tag{S3}$$

where r_A^i and r_B^i are vectors with the Cartesian coordinates of atom i of the molecules A and B, respectively, while $\| \cdot \|$ represents the Euclidean norm. To avoid the lack of invariance of the Cartesian coordinates with respect to rotation and permutation of equivalent atoms, we have employed a superimposing algorithm available in the literature.[8]

S-2.5 Effective Coordination Concept

The effective coordination number (ECN) and the local coordination number (LCN) were calculated to characterize the adsorption sites in the clusters. LCN is simply the number of atoms directly bonded to a specific one. To capture differences in the coordination environment of an atom i caused by different bond lengths, the ECN can be employed and is given as[9–11]

$$\mathrm{ECN}^i = \sum_j \exp\left\{\left[1 - \left(\frac{d_{ij}}{d_{av}^i}\right)^6\right]\right\}\,, \tag{S4}$$

where d_{ij} denotes the distance between atoms i and j, and d_{av}^i is determined through a self-consistent process as[9]

$$d_{av}^{i,new} = \frac{\sum_j d_{ij} \exp\left[1 - \left(\frac{d_{ij}}{d_{av}^{i,old}}\right)^6\right]}{\sum_j \exp\left[1 - \left(\frac{d_{ij}}{d_{av}^{i,old}}\right)^6\right]}. \tag{S5}$$

Furthermore, we also calculate the average ECN of the atoms comprising the adsorption site, ENC_{av}:

$$\mathrm{ENC}_{av} = \frac{1}{N_b} \sum_i^{N_b} \mathrm{ENC}^i\,, \tag{S6}$$

where N_b is the number of atoms to which atomic H is bonded.

S-2.6 Number of Adsorption Sites and Generation of Adsorption Structures

We counted the number of possible non-equivalent (by symmetry) adsorption sites (*CS*) for each cluster size and composition. For this, we have used the numbers LCN and ECN as guides, as exemplified for the unary group Ni_{13} in Table S-3 and Figure S-1. This counting is performed to estimate the number of possible adsorption configurations, on which we aim to perform DFT optimization later. To guarantee that all sites will be explored at the DFT level, we request that the algorithms that generate the adsorbed configurations provide 25 % more configurations than the value *CS*, which is denoted as *Ad* in Table S-4.

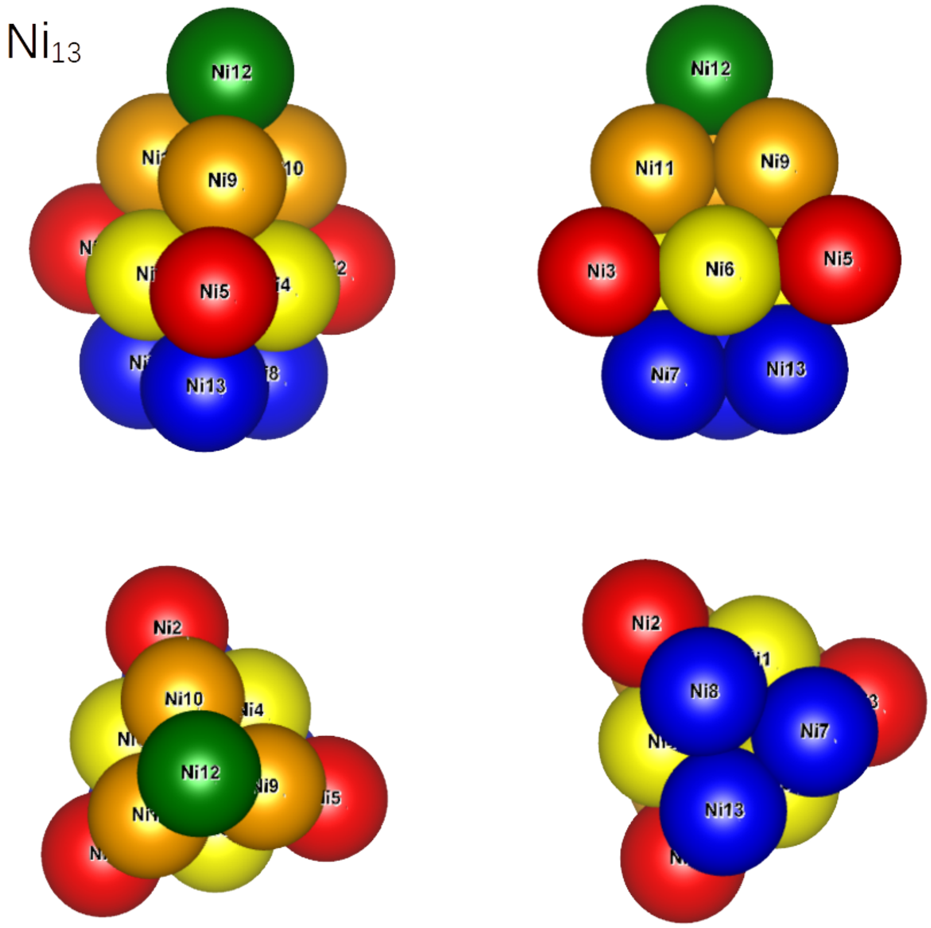

Figure S-1: The Ni_{13} cluster shown in three different orientations, with equivalent atoms colored similarly.

Table S-3: Effective coordination number (ECN) and local coordination number (LCN), in number of nearest neighbors (NNN), for each labeled atom for the Ni_{13} cluster.

Ni atom label	ECN^i (NNN)	LCN^i (NNN)
1	7.9	8
2	4	4
3	4	4
4	7.9	8
5	4	4
6	7.9	8
7	5	5
8	5	5
9	5.9	6
10	5.9	6
11	5.9	6
12	3	3
13	5	5

Table S-4: Counted sites (CS) and numbers adjusted by the 25 % correction factor (*Ad*) for each cluster.

Cluster	CS	*Ad*	Cluster	CS	*Ad*
Pd_{13}	30	38	Pd_{27}	52	65
Ni_1Pd_{12}	42	53	Ni_3Pd_{24}	122	153
Ni_3Pd_{10}	42	53	Ni_7Pd_{20}	127	159
Ni_5Pd_8	41	51	$Ni_{10}Pd_{17}$	124	155
Ni_7Pd_6	68	85	$Ni_{14}Pd_{13}$	120	150
Ni_8Pd_5	41	51	$Ni_{17}Pd_{10}$	124	155
$Ni_{10}Pd_3$	58	73	$Ni_{20}Pd_7$	123	154
$Ni_{12}Pd_1$	33	41	$Ni_{24}Pd_3$	110	138
Ni_{13}	19	24	Ni_{27}	48	60

S-3 Additional Data and Complementary Analysis

S-3.1 Selection of Representative Structures for the High-Cost Optimization Calculations

The total number of calculations performed on each step is summarized in Table S-5.

Table S-5: Number of Screening and High-cost Optimization Calculations for Each Cluster.

Cluster	Screening	Final	Cluster	Screening	Final
Pd_{13}	38	8	Pd_{27}	65	22
Ni_1Pd_{12}	53	11	Ni_3Pd_{24}	153	32
Ni_3Pd_{10}	53	11	Ni_7Pd_{20}	159	47
Ni_5Pd_8	51	1	$Ni_{10}Pd_{17}$	155	22
Ni_7Pd_6	85	2	$Ni_{14}Pd_{13}$	150	21
Ni_8Pd_5	51	1	$Ni_{17}Pd_{10}$	155	30
$Ni_{10}Pd_3$	73	3	$Ni_{20}Pd_7$	154	30
$Ni_{12}Pd_1$	41	1	$Ni_{24}Pd_3$	138	30
Ni_{13}	24	1	Ni_{27}	60	33

S-3.2 13-atoms Unary and Binary NiPd Clusters

S-3.2.1 Low-Cost Screening Optimization Calculations

The results of the electronic properties of all DFT geometry optimizations performed at the screening level are given in Figure S-2. Starting with the magnetic moment, we highlight that the adsorption of a hydrogen atom can affect m_{tot}. It is known that the adsorption of molecules often reduce the TM_{13} total magnetic moment, as observed for the adsorption of CO_2, COOH, H, CO, OH and O on Fe_{13} and Co_{13}.[12] On other hand, molecules like CO and O did not influence on m_{tot} of Ni clusters, while the adsorption of COOH and OH contribute to increase m_{tot} on such clusters.[12,13] Figure S-2 shows that, in our case, m_{tot} can change by one unit or remain constant.

The adsorption energies for all structures are substantially negative, reaching below -3 eV, as shown in Figure S-2. The strongest adsorption was observed for lower Ni content (Ni_1Pd_{12}). The graph for ΔE_{tot} shows that the relative energies are well distributed, which is the result of the selection of a diverse set of structures by the clustering algorithm.

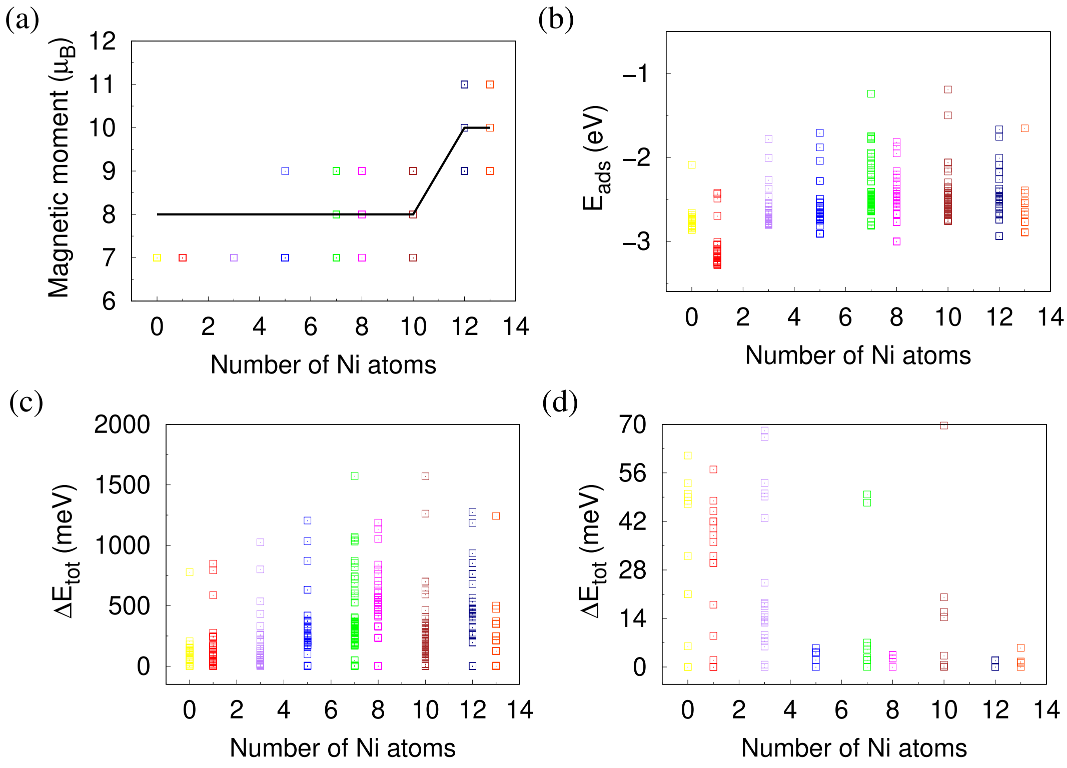

Figure S-2: Energetic and magnetic properties for $Ni_mPd_{13-m}-H$ systems as function of Ni content at the screening DFT level. Panel (a) shows the magnetic moment, with the black line providing the value of the bare cluster (without the hydrogen atom). Panel (b) shows the adsorption energy E_{ads}, while panel (c) provides the relative energies for all configurations. Panel (d) focuses only on the range of ΔE_{tot} that are selected for the high-cost optimization calculations.

S-3.2.2 High-Cost Optimization Calculations

After the high-cost optimization calculations, we observed that m_{tot} decreased for most compositions (compared to non-adsorbed structures), as shown in Figure S-3 (a). Panel (b) of this figure shows that the strongest adsorption shifted from Ni_1Pd_{12} at the screening level to Ni_3Pd_{10} at the final one. It is also seen that the adsorption strength tends to be higher in the alloys than in the unary clusters. The lowest energy configuration for each composition, shown in Figure S-3(c), was chosen for the harmonic frequency calculations.

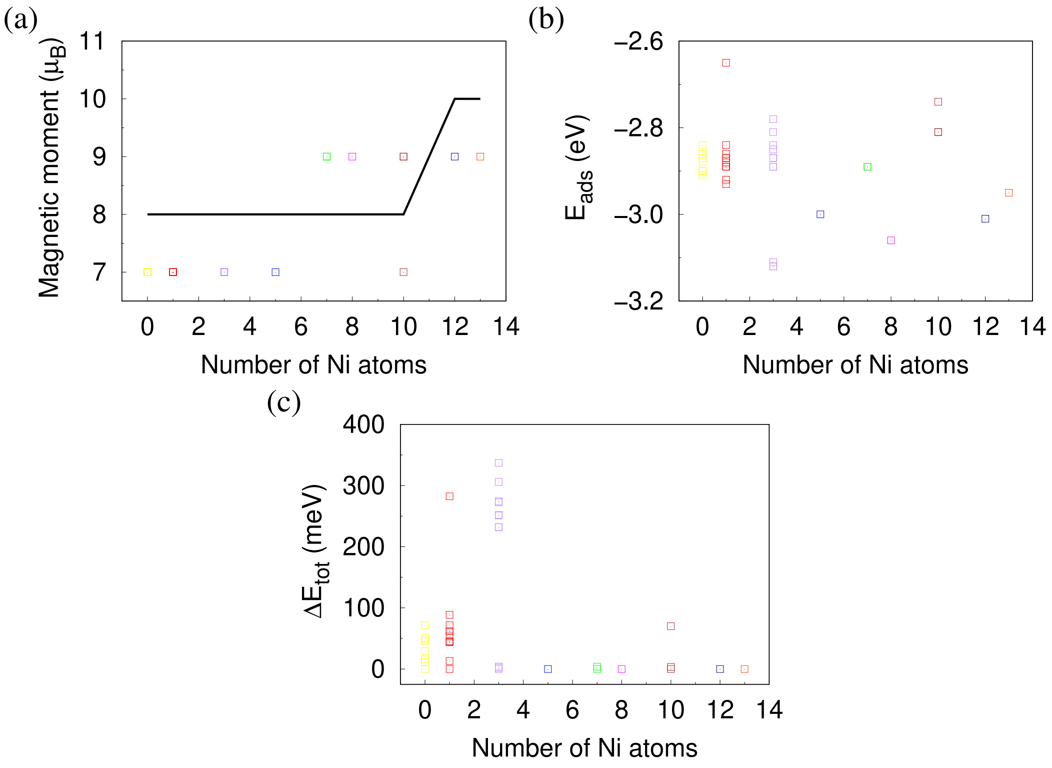

Figure S-3: Energetic and magnetic properties for $Ni_mPd_{13-m}-H$ systems at the final DFT level. Panel (a) shows the magnetic moment, with the black line providing the value of the bare cluster (without the hydrogen atom). Panel (b) shows the adsorption energy E_{ads}, while panel (c) provides the relative energies for all configurations.

S-3.2.3 Structural Parameters and Free Energy

Figure S-4 shows the lowest energy structure for each composition with $n = 13$, whereas other geometric parameters are given in Table S-6. It can be seen that H adsorption has a preference for bridge and hollow sites, at the PBE level. This is also the case for hydrogen on $Cu_{55-n}M_n$ (M =Co, Ni, Ru and Rh), where bridge and hollow sites were more energetically favored.[14]

Experimentally, the distance from an adsorbed hydrogen atom to the surface has been reported to be 1.72 Å ± 0.01 for three-fold coordinated sites in Ni (110)[15] using LEED structural analysis. For Pd(111) surface, the experimental measurement is 1.78 Å ± 0.05 Å.[16,17] As seen in Table S-6, which discriminates distances PdH and NiH separately, our calculated values are in agreement with the measured ones. This table also provides the ECN of the atoms of the cluster that compose the adsorption site and their average (ECN_{av}), which varies from 4.31 NNN to 5.50 NNN.

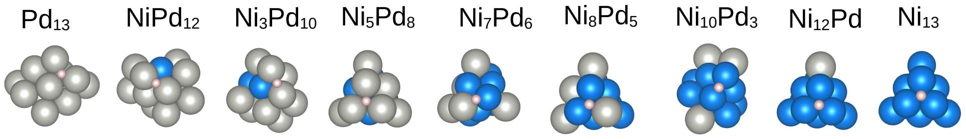

Figure S-4: Lowest energy configurations for hydrogen adsorption on Ni_mPd_{13-m}. Gray represents palladium atoms, nickel in blue and hydrogen in pink.

Table S-6: For each atom of the cluster that compose the site of adsorption, it is provided the distance to the H atom ($d_{\mathrm{H}-atom}$ in Å), average distance ($d_{average}$ in Å), ECN before adsorption (in number of nearest neighbors, NNN), and average ECN (ECN_{av}) for Ni_mPd_{13-m} clusters.

Cluster	atom	$d_{\mathrm{H}-atom}$	$d_{average}$	ECN	ECN_{av}
Pd_{13}	Pd	1.764	1.810	3.99	5.50
	Pd	1.800		4.96	
	Pd	1.865		7.53	
$NiPd_{12}$	Pd	1.737	1.711	6.72	5.32
	Pd	1.684		3.93	
Ni_3Pd_{10}	Pd	1.706	1.713	4.91	4.31
	Pd	1.721		3.71	
Ni_5Pd_8	Pd	1.773	1.811	4.89	4.92
	Pd	1.866		4.87	
	Pd	1.795		4.98	
Ni_7Pd_6	Ni	1.677	1.704	4.80	4.31
	Pd	1.730		3.81	
Ni_8Pd_5	Pd	1.933	1.792	5.00	4.88
	Ni	1.723		4.82	
	Ni	1.720		4.81	
$Ni_{10}Pd_3$	Ni	1.612	1.602	6.80	5.32
	Ni	1.592		3.84	
$Ni_{12}Pd$	Ni	1.755	1.736	4.98	4.93
	Ni	1.719		4.84	
	Ni	1.735		4.98	
Ni_{13}	Ni	1.744	1.737	4.98	4.98
	Ni	1.728		4.98	
	Ni	1.740		4.98	

Figure S-5 shows the results for the Gibbs free energy involved in the hydrogen evolution reaction steps. We can notice that the Ni_{13}, Pd_{13}, $NiPd_{12}$ and Ni_7Pd_6 had ΔG between -0.4 eV

and −0.5 eV, these values are close the ones observed for adsorption step for HER on Ru nanoclusters embedded in N, S co-doped carbonaceous shell[18] and the hydrogen adsorption on face (hollow site) of Pd_4 supported on VS_2 surface, where ΔG was −0.48 eV.[19] For comparison the Gibbs free energies for nickel surfaces are[20] −0.312 eV, −0.169 eV, and −0.32 eV for Ni(100), Ni(110), and Ni(111), respectively.

For palladium, the values are −0.32 eV, −0.254 eV, and −0.342 eV for Pd(100), Pd(110), and Pd(111), respectively.[20] Furthermore, we obtained Gibbs energies lower than −0.5 eV for some alloys. These cases strongly bind hydrogen atoms, hindering desorption.[21] In fact, the optimal value of ΔG would be as close to zero as possible.[22,23] Interestingly, $Ni_{10}Pd_3$ shows a more promising result, which is less negative than either of the unary clusters.

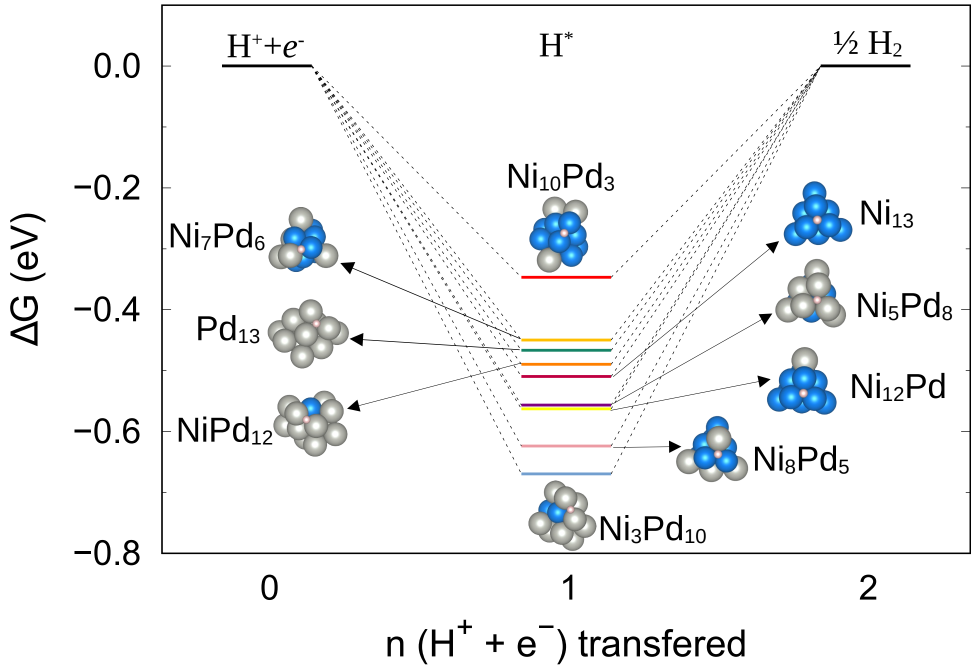

Figure S-5: Gibbs diagram for HER on clusters surfaces and adsorbed clusters. The pink spheres are the hydrogen atoms, blue ones are the nickel atoms and the gray are the palladium atoms.

As shown in Figure S-6, we can note that the highest ΔG was obtained in the lowest configurations ECN^H, but there is no clear correlation between the two properties.

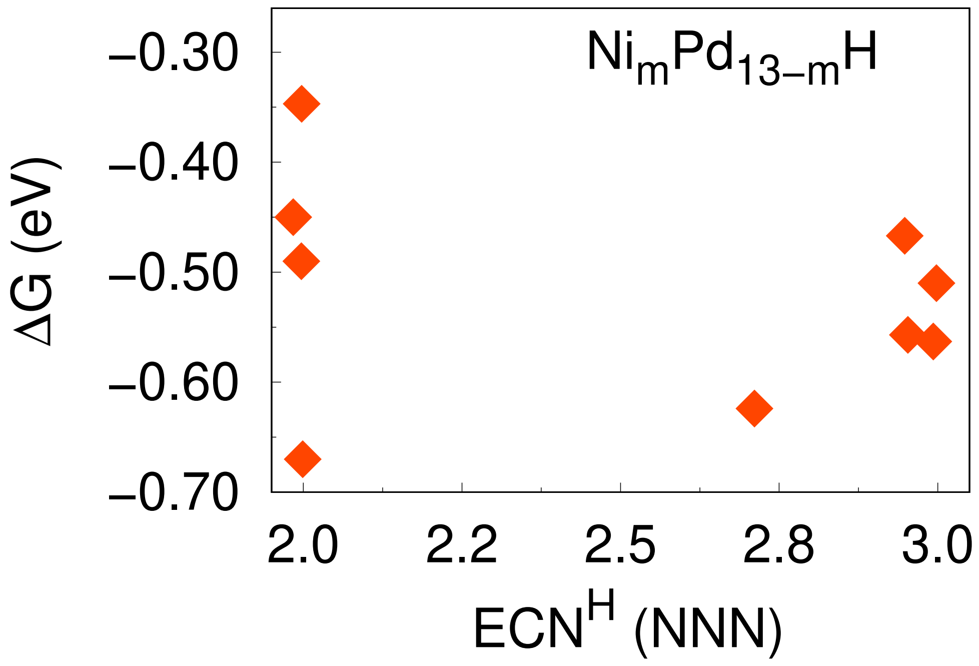

Figure S-6: ΔG as function of ECN^{H}.

S-3.3 27-atoms Unary and Binary NiPd Clusters

S-3.3.1 Low-Cost Screening Optimization Calculations

The magnetic effects of hydrogen adsorption on the surface, particularly for low-cost screening optimization calculations, were less pronounced for the $\mathrm{Ni}_m\mathrm{Pd}_{27-m}\mathrm{H}$ system compared to the $\mathrm{Ni}_m\mathrm{Pd}_{13-m}\mathrm{H}$, as shown in Figure S-7(a). Among the former, only Pd_{27} exhibited a significant variation in the total magnetization (m_{tot}). Ref. 17 reported a reduction in m_{tot} after hydrogen adsorption on the Ni(111), Co(0001), and Fe(110) surfaces.[17] In fact, as we increase the system size from 13 to 27 atoms, we observe that clusters, particularly Ni_{27}, tend to exhibit magnetic moments closer to those found in bulk materials.

The adsorption energies of bulk fcc structures of Ni and Pd were 2.71 eV and 2.68 eV, respectively.[17] For the $\mathrm{Ni}_m\mathrm{Pd}_{27-m}\mathrm{H}$ configurations, the majority of adsorption energies (E_{ads}) ranged from -2 to -3 eV, as shown in Figure S-7(b). Figure S-7(c) present ΔE_{tot} for all configurations while S-7(d) focus on the selected for the next step (high-cost optimization calculations), which must lie below 140 meV.

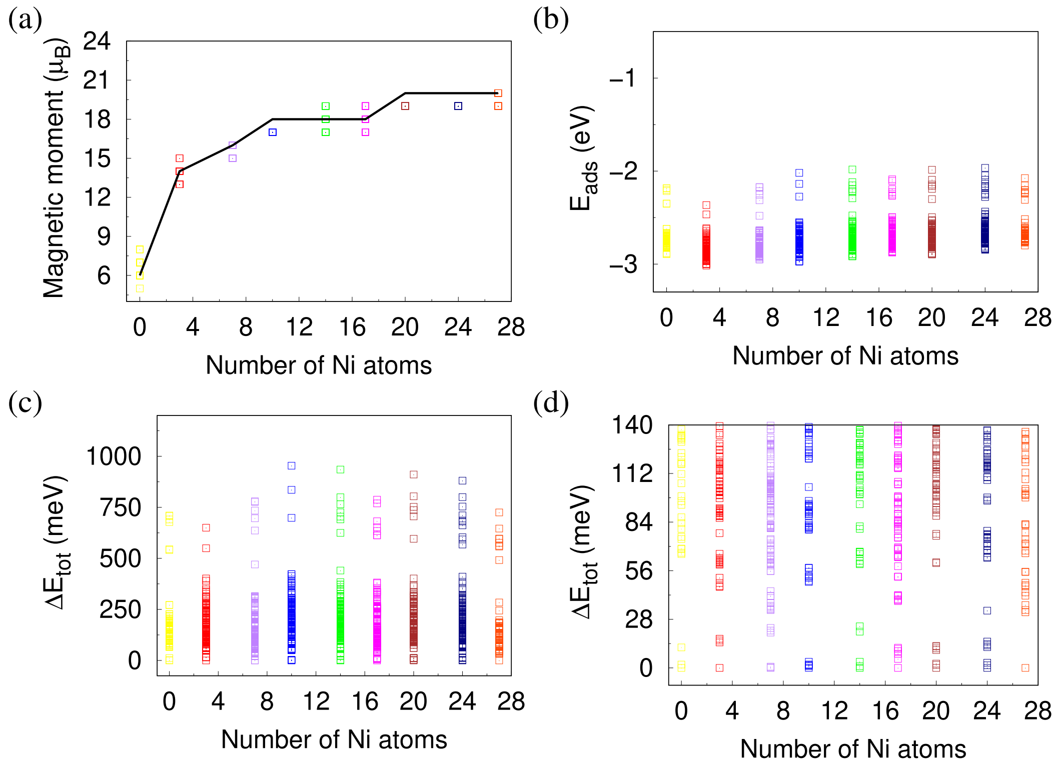

Figure S-7: Energetic and magnetic properties for $Ni_mPd_{27-m}-H$ systems as function of Ni content at the screening DFT level. Panel (a) shows the magnetic moment, with the black line providing the value of the bare cluster (without the hydrogen atom). Panel (b) shows the adsorption energy E_{ads}, while panel (c) provides the relative energies for all configurations. Panel (d) focuses only on the range of ΔE_{tot} that are selected for the high-cost optimization calculations.

S-3.3.2 High-Cost Optimization Calculations

As observed in Ref. 24, clusters tend to exhibit exceptional magnetic properties, which are promoted by their reduced size and the spin-orbit coupling (SOC) effect. These effects lead to intricate interactions that influence the magnetic moment.[24] Ferromagnetic transition metals Fe, Co, and Ni are the primary focus of experimental studies, as noted by Ref. 25, which discusses the total magnetization (m_{tot}) of Ni alloys. The m_{tot} is influenced by both the atomic connectivity and the shape of the clusters.[25] Despite Pd not being an inherently magnetic element, small Pd clusters tend to present a magnetic moment.[26] In fact, as we noted, Pd_{13} presents a significant m_{tot},[27] but $m_{tot} = 0$ for Pd_{27}, implying that the geometry of the cluster strongly influences m_{tot} due to the significant structural difference between these two clusters.

As shown in Figure S-8 (a), groups with more than ten Ni atoms generally follow the same trend observed in the low-cost screening optimization calculations, while groups with fewer Ni

exhibit greater variations in their m_{tot}, particularly for Ni_3Pd_{24}, where m_{tot} varies from $0\mu_B$ to 16 μ_B. Recall that only in the high-cost optimization calculations is the cluster allowed to relax, and interactions of H atoms with the cluster can promote distortions in geometry that further change the m_{tot} of the configurations.

Figures S-8(b) and S-8(c) show the adsorption energy (E_{ads}) and the total change in energy (ΔE_{tot}) as a function of the number of Ni atoms, respectively. It is evident that these parameters exhibit greater variation for clusters with fewer than 10 nickel atoms. Therefore, these properties are likely correlated with the total magnetic moment (m_{tot}).

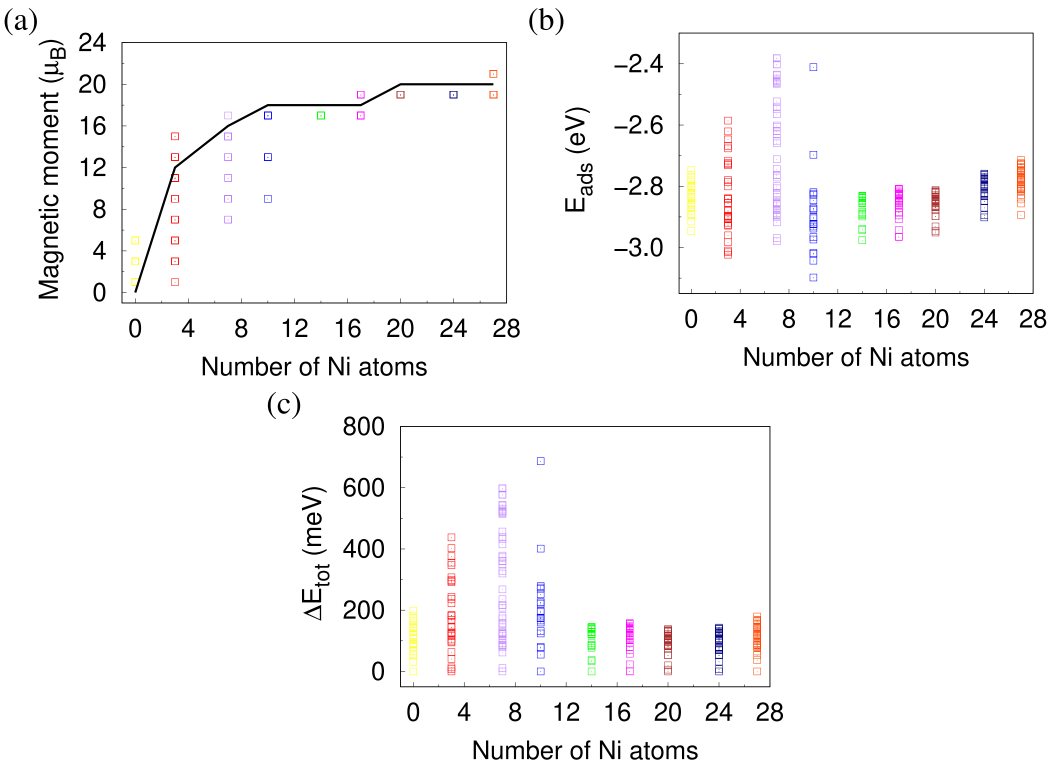

Figure S-8: Energetic and magnetic properties for $Ni_mPd_{27-m}-H$ systems at the final DFT level. Panel (a) shows the magnetic moment, with the black line providing the value of the bare cluster (without the hydrogen atom). Panel (b) shows the adsorption energy E_{ads}, while panel (c) provides the relative energies for all configurations.

S-3.3.3 Structural Parameters and Free Energy

As shown in Figure S-9, for all lowest-energy $Ni_mPd_{27-m}H$ configurations, the hydrogen adsorbs on hollow sites. These results are different from those observed for the Ni_mPd_{13-m} clusters, where hydrogen also adsorbs on the bridge positions.

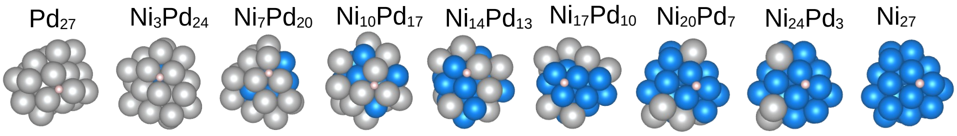

Figure S-9: Lowest energy configurations for hydrogen adsorption on Ni_mPd_{27-m}. Gray represents palladium atoms, nickel in blue and hydrogen in pink.

For the 27-atom system, we notice that the d_{av} for the unary clusters is 1.81 Å for Pd_{27} and 1.72 Å for Ni_{27}, as shown in Table S-7. This is also in agreement with the experimental values mentioned earlier.[15–17] For alloys, the distance between H and the closest atoms of the cluster depends on the atomic species to which hydrogen is bound. However, in general, $d_{average}$ shows a strong dependence on composition, with Ni rich clusters having the lowest average distance. As expected, the ECN_{av} of the atoms to which H bonds is greater than that of the 13-atom systems, as shown in Table S-7.

Table S-7: For each atom of the cluster that compose the site of adsorption, it is provided the distance to the H atom (d_{H-atom} in Å), average distance ($d_{average}$ in Å), ECN before adsorption (in number of nearest neighbors, NNN), and average ECN (ECN_{av}) for Ni_mPd_{13-m} clusters.

Cluster	atom	d_{H-atom}	$d_{average}$	ECN	ECN_{av}
Pd_{27}	Pd	1.861	1.813	6.94	6.26
	Pd	1.802		6.89	
	Pd	1.775		4.94	
Ni_3Pd_{24}	Pd	1.890	1.824	7.60	6.98
	Pd	1.775		6.74	
	Pd	1.808		6.60	
Ni_7Pd_{20}	Pd	1.823	1.822	7.51	6.65
	Pd	1.809		5.71	
	Pd	1.833		6.74	
$Ni_{10}Pd_{17}$	Pd	1.788	1.836	5.86	5.97
	Pd	1.913		6.35	
	Pd	1.807		5.71	
$Ni_{14}Pd_{13}$	Pd	1.827	1.761	7.15	6.78
	Ni	1.765		7.44	
	Ni	1.692		5.77	
$Ni_{17}Pd_{10}$	Ni	1.740	1.718	6.22	5.85
	Ni	1.747		6.54	
	Ni	1.668		4.80	
$Ni_{20}Pd_7$	Ni	1.697	1.720	5.95	6.91
	Ni	1.732		7.27	
	Ni	1.730		7.51	
$Ni_{24}Pd_3$	Ni	1.682	1.716	5.90	7.03
	Ni	1.729		7.54	
	Ni	1.737		7.65	
Ni_{27}	Ni	1.681	1.723	5.86	7.06
	Ni	1.744		7.67	
	Ni	1.744		7.66	

Figure S-10 shows the Gibbs free energy results for the Ni_mPd_{27-m} clusters. By comparing ΔG values between the Ni_mPd_{27-m} and Ni_mPd_{13-m} systems, we notice that the highest ΔG for the 13-atom system is 0.09 eV higher. The values of ΔG near 0 are interesting due to the reduction of overpotential. In volcano plot representations, we can always see that the values

ΔG correspond to the lowest overpotential, which means that we need a small current density to drive this reaction.[23,28] Here, we can see that the unary Pd and Ni are more promising catalysts from HER than the alloys, unlike those obtained for the smaller clusters. However, $Ni_{24}Pd_3$ has a ΔG similar to the unary ones.

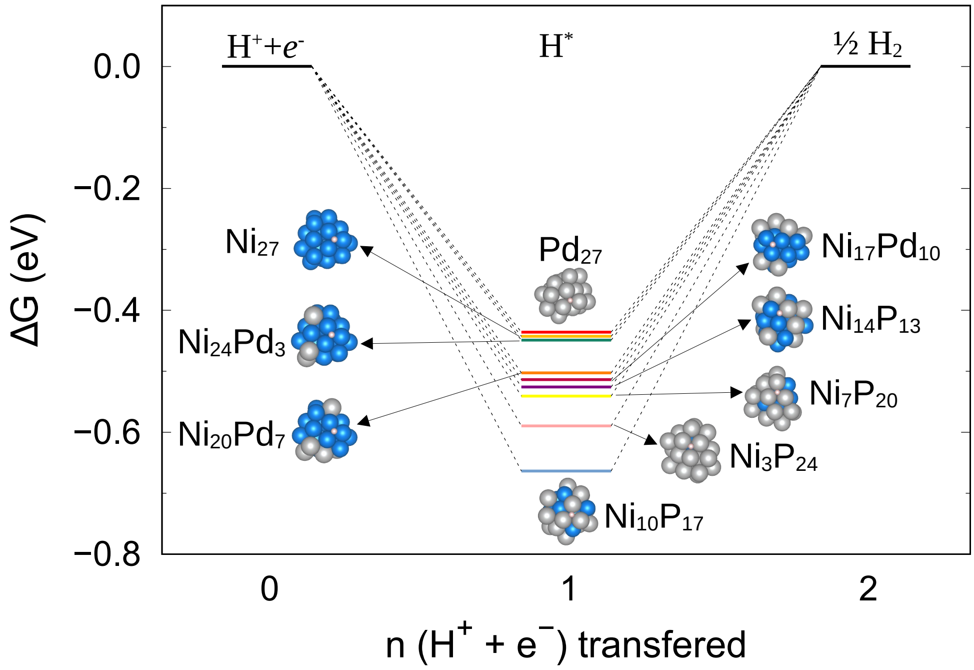

Figure S-10: Gibbs diagram for HER on clusters surfaces and adsorbed clusters. The pink spheres are the hydrogen atoms, blue ones are the nickel atoms and the gray are the palladium atoms.

The relationship between ECN^{H} and ΔG for the 27 atom groups is clearer than for the 13 atom groups, with the highest ΔG occurring at ECN^{H} values between 2.96 NNN and 2.98 NNN, as shown in Figure S-11. If we exclude Pd_{27}, we observe similar trends to those for Ni_mPd_{13-m}, where lower ΔG configurations tend to have higher $d_{average}$, while a higher ΔG is associated with a low average distance between the hydrogen atom and the cluster.

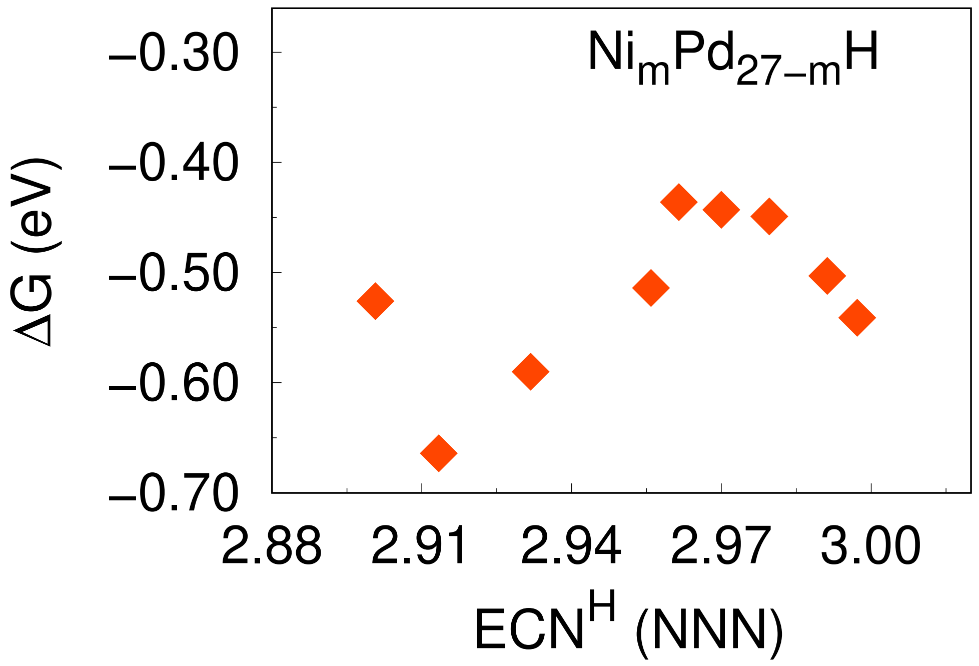

Figure S-11: ΔG as function of ECN^{H}.

S-4 Tables With Cartesian Coordinates and Net Charge Distribution of Non-Adsorbed Configurations

We perform Density Derived Electrostatic and Chemical (DDEC6) analysis for understanding electronic density distribution between cluster atoms. Atoms with a great electronic density show a more negative charge value since the charge of the average electron number overcomes their nuclei charges; thus, the difference between the charges of the electron cloud and the nuclei give us the partial net charge for each cluster atoms. The following tables present the charge distribution in all the more stable cluster before and after hydrogen adsorption.

S-4.1 13-atoms Unary and Binary NiPd Clusters

Table S-8: Atomic coordinates and respective DDEC6 partial net charges of each atom in Pd_{13}.

Atom	x (Å)	y (Å)	z (Å)	Partial Net Charge (e)
Pd	10.993248	11.572302	10.629318	−0.016265
Pd	10.139283	12.581940	8.361024	0.004147
Pd	10.068198	9.955512	8.383011	−0.017902
Pd	10.265346	7.464639	9.134979	0.002941
Pd	12.418728	11.181933	8.451072	0.014026
Pd	12.132624	9.118473	10.242561	−0.011628
Pd	8.366862	11.367636	9.902445	−0.011542
Pd	7.857654	8.773086	9.226266	0.002862
Pd	9.187815	11.279751	12.522510	0.014210
Pd	7.124040	9.810423	11.607435	0.016481
Pd	11.532171	9.999066	12.661824	0.004238
Pd	9.606954	9.055557	11.142663	−0.017791
Pd	12.222651	8.611722	7.671930	0.016224

Table S-9: Atomic coordinates and respective DDEC6 partial net charges of each atom in $NiPd_{12}$.

Atom	x (Å)	y (Å)	z (Å)	Partial Net Charge (e)
Ni	10.881066	11.373999	10.542441	−0.045154
Pd	10.159002	12.602289	8.483475	0.007886
Pd	10.058916	9.953475	8.378496	−0.012615
Pd	10.267173	7.460691	9.119355	−0.001890
Pd	12.394935	11.189976	8.559390	0.027641
Pd	12.136845	9.100434	10.232019	−0.004749
Pd	8.344119	11.363100	9.896565	−0.003303
Pd	7.844613	8.771511	9.230214	−0.000320
Pd	9.239244	11.329458	12.430551	0.028377
Pd	7.112406	9.810444	11.595801	0.004260
Pd	11.569950	10.091655	12.581478	0.007906
Pd	9.605841	9.047850	11.135922	−0.011707
Pd	12.222252	8.597925	7.672182	0.003668

Table S-10: Atomic coordinates and respective DDEC6 partial net charges of each atom in Ni_3Pd_{10}.

Atom	x (Å)	y (Å)	z (Å)	Partial Net Charge (e)
Ni	11.872203	9.221877	10.137204	−0.035658
Ni	8.565396	11.144427	9.963996	−0.036063
Ni	10.890915	11.383932	10.552332	−0.053222
Pd	10.018134	12.588198	8.593410	0.026409
Pd	10.083969	9.979830	8.305626	−0.015655
Pd	10.277799	7.494060	9.163917	0.003857
Pd	12.412680	11.221266	8.616258	0.029054
Pd	7.883274	8.805237	9.231831	0.003628
Pd	9.278367	11.382546	12.420366	0.029334
Pd	7.107849	9.955638	11.513796	0.019009
Pd	11.683686	10.039386	12.453714	0.026312
Pd	9.594123	9.029979	11.225865	−0.015594
Pd	12.312972	8.591457	7.824558	0.018588

Table S-11: Atomic coordinates and respective DDEC6 partial net charges of each atom in Ni_5Pd_8.

Atom	x (Å)	y (Å)	z (Å)	Partial Net Charge (e)
Ni	10.769682	9.163854	8.626947	−0.026215
Ni	9.853368	9.733395	10.722915	−0.021257
Ni	12.194532	10.321290	10.277043	−0.003646
Ni	11.608611	11.458083	8.282064	−0.013966
Ni	10.583328	12.062715	10.422258	−0.013770
Pd	9.103857	11.175255	8.552691	0.004733
Pd	10.476543	10.384311	6.438327	0.008665
Pd	8.282883	11.677386	11.026512	0.004123
Pd	11.690994	8.013957	10.677954	0.011168
Pd	9.273726	7.466991	9.710169	0.015718
Pd	7.478415	9.421587	9.815736	−0.003517
Pd	8.566887	8.758071	7.456701	0.002369
Pd	12.907734	12.618228	9.974286	0.035595

Table S-12: Atomic coordinates and respective DDEC6 partial net charges of each atom in Ni_7Pd_6.

Atom	x (Å)	y (Å)	z (Å)	Partial Net Charge (e)
Ni	10.029873	9.839613	8.513547	−0.071403
Ni	12.032916	10.946607	8.506134	0.037603
Ni	11.818968	9.140943	10.058076	−0.023134
Ni	8.480241	11.108916	9.809940	−0.030744
Ni	7.992180	8.806602	9.203712	0.029100
Ni	10.746162	11.312889	10.442061	−0.064882
Ni	9.182838	11.002110	12.175968	0.035651
Pd	9.975126	12.372780	8.318289	0.040122
Pd	10.016034	7.379442	8.911644	−0.008055
Pd	7.029939	9.837198	11.311314	0.023381
Pd	11.482758	10.034325	12.415578	0.014547
Pd	9.516969	8.861832	11.077983	−0.018041
Pd	12.090666	8.607879	7.671867	0.035855

Table S-13: Atomic coordinates and respective DDEC6 partial net charges of each atom in Ni_8Pd_5.

Atom	x (Å)	y (Å)	z (Å)	Partial Net Charge (e)
Ni	9.207534	10.929093	8.722518	−0.046365
Ni	8.326941	11.576502	10.780392	0.050347
Ni	9.812964	9.784740	10.674573	−0.046188
Ni	9.291618	7.723317	9.617874	0.007797
Ni	8.687805	8.861307	7.678188	0.007814
Ni	12.143418	10.273221	10.281264	−0.014450
Ni	11.517513	11.454975	8.266692	−0.014167
Ni	10.510710	12.062736	10.387356	−0.019228
Pd	10.163538	10.481709	6.441834	0.012220
Pd	11.108076	8.936403	8.368500	0.002623
Pd	11.510730	7.936551	10.783857	0.011762
Pd	7.449414	9.437400	9.798873	0.025606
Pd	12.845700	12.582591	9.967041	0.022229

Table S-14: Atomic coordinates and respective DDEC6 partial net charges of each atom in $Ni_{10}Pd_3$.

Atom	x (Å)	y (Å)	z (Å)	Partial Net Charge (e)
Ni	9.960300	12.119667	8.359491	0.082175
Ni	9.912966	9.794148	8.374758	−0.089614
Ni	10.047933	7.601832	9.047619	0.043189
Ni	11.953746	10.879071	8.422113	0.042787
Ni	11.735829	9.036468	10.009020	−0.058540
Ni	8.394939	11.013996	9.727872	−0.036737
Ni	7.975086	8.706873	9.115743	0.030758
Ni	10.693641	11.192748	10.360581	−0.075853
Ni	11.254047	9.788457	12.129810	0.068651
Ni	9.480030	9.044301	10.834236	−0.061122
Pd	9.107490	11.087139	12.273576	0.016926
Pd	7.033446	9.600822	11.229456	0.009569
Pd	11.932473	8.472933	7.619220	0.027811

Table S-15: Atomic coordinates and respective DDEC6 partial net charges of each atom in $Ni_{12}Pd_1$

Atom	x (Å)	y (Å)	z (Å)	Partial Net Charge (e)
Ni	9.057111	10.782828	8.505210	−0.031990
Ni	10.110324	10.200120	6.538224	0.048074
Ni	10.691268	8.987685	8.420349	−0.040596
Ni	11.301717	7.939743	10.389099	0.047384
Ni	9.693579	9.579129	10.555944	−0.032276
Ni	9.126915	7.541268	9.458778	0.002181
Ni	7.551264	9.248715	9.546117	0.004760
Ni	8.523711	8.687091	7.508172	0.001864
Ni	12.019917	10.144176	10.104255	−0.007319
Ni	11.408502	11.298735	8.133720	−0.008370
Ni	10.443405	11.897760	10.203648	−0.006089
Ni	12.648699	12.333216	9.770481	0.015406
Pd	8.056860	11.457390	10.692297	0.006971

Table S-16: Atomic coordinates and respective DDEC6 partial net charges of each atom in Ni_{13}.

Atom	x (Å)	y (Å)	z (Å)	Partial Net Charge (e)
Ni	9.045897	10.663065	8.837997	−0.032467
Ni	10.099929	10.092999	6.851628	0.043315
Ni	8.177316	11.234727	10.907442	0.042437
Ni	10.690575	8.874243	8.734026	−0.031735
Ni	11.291637	7.827078	10.712247	0.042391
Ni	9.673671	9.473436	10.865862	−0.033060
Ni	9.116037	7.439817	9.775542	−0.000402
Ni	7.539084	9.161817	9.878400	0.000232
Ni	8.516088	8.586165	7.829472	−0.000566
Ni	12.002340	10.027815	10.421607	−0.012863
Ni	11.389161	11.184432	8.447502	−0.014682
Ni	10.409805	11.770416	10.518396	−0.012612
Ni	12.628287	12.217254	10.082688	0.010012

S-4.2 27-atoms Unary and Binary NiPd Clusters

Table S-17: Atomic coordinates and respective DDEC6 partial net charges of each atom in Pd_{27}.

Atom	x (Å)	y (Å)	z (Å)	Partial Net Charge (e)
Pd	7.710390	11.273602	13.253602	0.033213
Pd	8.100472	9.270067	11.388285	−0.028476
Pd	8.536027	11.907945	10.875870	−0.049002
Pd	8.562847	10.323045	8.708017	−0.028446
Pd	8.650192	8.932095	14.068282	−0.006595
Pd	9.058612	13.076955	8.645535	0.047280
Pd	9.586778	11.171070	15.131137	−0.010890
Pd	9.719370	13.025565	13.137727	−0.032525
Pd	10.063103	11.565090	6.761610	−0.007247
Pd	10.182532	7.669080	12.355380	0.046522
Pd	10.309567	10.546560	12.278745	0.112684
Pd	10.302053	8.659080	9.980775	−0.049174
Pd	10.688962	13.515165	10.688872	−0.047129
Pd	10.725637	9.097627	7.457287	0.033180
Pd	11.034337	11.132865	9.467392	0.112660
Pd	11.363220	9.229185	14.303722	−0.031255
Pd	11.754562	13.253467	8.077342	−0.031183
Pd	11.931120	11.846362	14.097532	−0.032149
Pd	12.160260	14.142757	12.778020	0.032632
Pd	12.389738	8.949577	11.661862	−0.047547
Pd	12.616425	10.898190	6.920460	−0.010253
Pd	12.800092	11.806335	11.625615	−0.001271
Pd	12.877222	9.225945	9.043875	−0.031588
Pd	13.189207	14.129595	10.239705	0.013602
Pd	13.716697	11.767725	9.167602	−0.032960
Pd	13.830277	10.117282	13.507807	0.013691
Pd	14.675985	10.003387	10.913647	0.032226

Table S-18: Atomic coordinates and respective DDEC6 partial net charges of each atom in Ni_3Pd_{24}.

Atom	x (Å)	y (Å)	z (Å)	Partial Net Charge (e)
Ni	10.804590	10.642095	9.565290	−0.025169
Ni	9.356355	8.843962	9.884362	−0.083957
Ni	11.054047	12.507435	11.221762	−0.018120
Pd	11.876692	10.998697	7.317270	0.021320
Pd	9.214110	10.273612	7.645320	0.040539
Pd	11.189722	8.493390	8.197492	0.035203
Pd	10.061527	12.738015	8.547255	−0.022299
Pd	12.748320	12.463380	9.396112	−0.003223
Pd	13.267620	9.796635	9.377685	−0.027606
Pd	8.048272	11.313990	9.761265	−0.027293
Pd	11.298150	14.741617	9.821452	0.010339
Pd	11.749725	7.756402	10.653930	−0.013157
Pd	8.983282	13.717282	10.863990	−0.025135
Pd	14.236222	11.419470	11.468655	−0.044954
Pd	13.431757	14.006880	11.397847	0.013992
Pd	7.310002	9.155520	11.276122	0.029998
Pd	14.185125	8.683447	11.619562	−0.003490
Pd	9.341415	10.608255	11.909227	0.068377
Pd	11.946172	10.151820	11.731162	0.137727
Pd	7.255417	12.151395	12.162960	0.010605
Pd	9.669622	8.090212	12.315983	−0.001386
Pd	11.063452	14.701747	12.521587	0.026571
Pd	12.141225	7.938360	13.336425	−0.028514
Pd	9.641272	12.660390	13.425367	−0.042452
Pd	12.372097	12.445312	13.334782	−0.017033
Pd	13.628655	10.178010	13.880115	−0.001441
Pd	10.866105	10.263667	14.107972	−0.009443

Table S-19: Atomic coordinates and respective DDEC6 partial net charges of each atom in Ni_7Pd_{20}.

Atom	x (Å)	y (Å)	z (Å)	Partial Net Charge (e)
Ni	13.000815	13.630410	11.299342	0.002061
Ni	13.638532	11.294617	11.325262	−0.038800
Ni	9.225427	8.705655	9.728280	−0.060624
Ni	10.750815	10.441170	9.575347	0.003830
Ni	11.045340	12.325815	11.107485	0.023725
Ni	9.383445	10.507117	11.821162	−0.031893
Ni	11.748037	10.041840	11.764755	0.006215
Pd	11.060145	8.340277	7.980232	0.016146
Pd	13.109625	9.751680	9.217755	−0.019497
Pd	9.532530	8.118877	12.237930	0.002265
Pd	14.008410	8.731732	11.456392	0.028545
Pd	11.763247	10.846980	7.170435	0.007645
Pd	7.286917	9.184297	11.306497	0.028340
Pd	7.246147	11.885107	12.069157	0.016683
Pd	8.199585	11.155612	9.726772	0.009678
Pd	12.252038	12.380220	13.347180	−0.021007
Pd	12.031425	7.866315	13.167945	−0.032018
Pd	10.079753	12.598110	8.429130	−0.038440
Pd	10.681087	10.138500	13.975312	0.038415
Pd	8.961097	13.557150	10.766070	−0.025682
Pd	10.997685	14.609520	12.442995	0.004123
Pd	11.279407	14.622525	9.723487	0.015352
Pd	9.150322	10.161360	7.506067	0.041999
Pd	9.563400	12.501495	13.269420	−0.013872
Pd	12.726180	12.379297	9.188325	0.015375
Pd	11.565967	7.881187	10.519605	−0.006071
Pd	13.502925	10.133392	13.667940	0.027509

Table S-20: Atomic coordinates and respective DDEC6 partial net charges of each atom in $Ni_{10}Pd_{17}$.

Atom	x (Å)	y (Å)	z (Å)	Partial Net Charge (e)
Ni	13.263975	10.095165	13.532175	−0.020658
Ni	9.772200	12.368160	13.003357	−0.081146
Ni	11.470545	8.113027	10.597185	−0.040549
Ni	11.632207	10.825042	7.553722	−0.016981
Ni	9.179955	13.337437	10.804950	−0.052527
Ni	8.341537	11.164500	9.816458	−0.053113
Ni	11.244960	12.308557	11.065995	0.037999
Ni	10.674877	10.444275	9.622530	0.021552
Ni	9.434745	10.435095	11.778210	−0.025937
Ni	11.813467	10.069222	11.737755	0.005692
Pd	14.041552	8.690040	11.536447	0.014770
Pd	9.092992	8.522190	9.665190	−0.028548
Pd	12.730837	12.474427	9.098887	−0.013239
Pd	10.705590	10.164487	13.970767	0.055043
Pd	13.074525	9.771435	9.329310	0.005281
Pd	11.199600	8.355825	8.007255	0.027718
Pd	11.109510	14.617417	9.796185	0.008134
Pd	13.227165	13.973670	11.358292	−0.022215
Pd	7.464037	12.048187	12.022177	0.098682
Pd	10.009125	12.614130	8.482072	0.012374
Pd	7.239015	9.390127	11.334915	0.006554
Pd	12.034485	7.889017	13.110952	0.010880
Pd	9.142267	10.234553	7.567875	0.034571
Pd	13.900590	11.402100	11.372895	−0.023297
Pd	12.248910	12.402405	13.404060	−0.001272
Pd	9.522698	8.070457	12.294382	−0.004622
Pd	10.762965	14.553360	12.470310	0.044854

Table S-21: Atomic coordinates and respective DDEC6 partial net charges of each atom in $Ni_{14}Pd_{13}$.

Atom	x (Å)	y (Å)	z (Å)	Partial Net Charge (e)
Ni	9.615668	12.404947	13.048537	−0.040762
Ni	12.835350	9.838350	9.317115	−0.041758
Ni	7.434630	9.320040	11.327310	0.014954
Ni	11.173027	14.139382	9.766868	−0.000416
Ni	10.138095	12.351577	8.618242	−0.059538
Ni	13.512870	11.252137	11.282557	−0.052367
Ni	11.065792	8.648122	8.068072	−0.006387
Ni	12.562695	12.232597	9.278392	−0.045250
Ni	9.348142	10.240830	7.628400	−0.010417
Ni	10.773180	10.235497	13.672147	0.023458
Ni	11.084422	12.192390	11.092230	0.047885
Ni	9.343125	10.541992	11.764012	−0.054793
Ni	11.693992	9.850365	11.627865	0.001752
Ni	10.530787	10.287135	9.615645	0.043032
Pd	13.934340	8.741902	11.261835	0.029825
Pd	7.270110	11.814187	12.105585	0.029906
Pd	11.796750	10.951065	7.257802	0.090522
Pd	8.914837	13.378725	10.622452	−0.008742
Pd	13.256887	13.738050	11.163240	0.050911
Pd	9.492142	8.178007	12.178665	0.016774
Pd	11.937487	7.882380	13.146367	−0.028223
Pd	8.919292	8.390092	9.487530	−0.019193
Pd	13.374315	10.132065	13.595625	0.016323
Pd	10.804545	14.511195	12.263287	0.011499
Pd	12.109793	12.420832	13.359982	−0.006161
Pd	11.505015	7.737997	10.330740	−0.011898
Pd	8.039835	11.055217	9.586597	0.009065

Table S-22: Atomic coordinates and respective DDEC6 partial net charges of each atom in $Ni_{17}Pd_{10}$.

Atom	x (Å)	y (Å)	z (Å)	Partial Net Charge (e)
Ni	11.516040	8.053020	10.510335	−0.038462
Ni	8.261932	11.098777	9.769005	−0.065705
Ni	12.902378	9.806377	9.397890	−0.057194
Ni	10.941525	14.159992	12.258855	−0.008746
Ni	9.190327	8.544667	9.681502	−0.036652
Ni	9.745470	8.479395	12.031785	−0.028003
Ni	11.900340	8.038710	12.881002	0.005610
Ni	11.595712	10.799460	7.590667	−0.011220
Ni	7.667010	9.342742	11.263747	0.064849
Ni	10.101465	12.415702	8.648482	−0.050091
Ni	13.671652	8.743590	11.404822	0.020630
Ni	13.008825	13.643977	11.301480	−0.001205
Ni	12.043777	12.153083	13.092727	−0.050842
Ni	11.774902	10.068187	11.730150	0.013127
Ni	11.156017	12.270285	10.919047	0.026153
Ni	10.616152	10.321042	9.640912	0.048487
Ni	9.417622	10.760850	11.815717	−0.043914
Pd	9.508410	12.578377	13.446832	0.001186
Pd	13.887045	11.307780	11.273670	0.004251
Pd	11.180610	8.343945	8.047912	0.048856
Pd	8.936257	13.395757	10.734030	0.011750
Pd	7.160445	11.767095	11.934022	0.024995
Pd	13.418842	10.006402	13.646857	0.004300
Pd	10.633612	10.063395	13.965052	0.038459
Pd	12.789112	12.402382	9.030307	0.021134
Pd	11.168280	14.522152	9.689805	0.005683
Pd	9.115290	10.221907	7.602457	0.052565

Table S-23: Atomic coordinates and respective DDEC6 partial net charges of each atom in $Ni_{20}Pd_7$.

Atom	x (Å)	y (Å)	z (Å)	Partial Net Charge (e)
Ni	11.689830	10.861267	7.508137	0.025484
Ni	9.350078	10.254285	7.607812	0.004902
Ni	11.095447	8.603325	8.050680	−0.006056
Ni	10.074352	12.371670	8.599432	−0.043191
Ni	12.544740	12.262297	9.197437	−0.039635
Ni	12.897967	9.840870	9.337635	−0.016521
Ni	10.617165	10.308442	9.526545	0.024646
Ni	9.027990	13.199175	10.747440	−0.039329
Ni	11.083680	12.148425	10.988010	0.044841
Ni	13.519530	11.275605	11.235240	−0.036734
Ni	12.972352	13.553482	11.249077	0.024483
Ni	9.368100	10.559722	11.611057	−0.043888
Ni	11.672730	9.863550	11.614747	0.000053
Ni	7.518105	11.849805	12.009262	0.027286
Ni	9.663097	8.361765	12.089407	−0.041960
Ni	10.838857	14.132160	12.226725	0.015616
Ni	9.601560	12.344310	13.049977	−0.017208
Ni	12.056310	12.207892	13.083615	−0.040523
Ni	13.143870	10.164015	13.378432	0.009328
Ni	10.663177	10.228838	13.640895	0.047271
Pd	8.025097	11.148435	9.577958	0.011515
Pd	8.954527	8.462520	9.535230	−0.009349
Pd	11.111872	14.398717	9.698962	0.054880
Pd	11.508412	7.724002	10.352587	−0.001814
Pd	7.293195	9.272047	11.560297	0.013698
Pd	13.929030	8.751352	11.402280	0.024775
Pd	11.862742	7.935817	13.204935	0.007430

Table S-24: Atomic coordinates and respective DDEC6 partial net charges of each atom in $Ni_{24}Pd_3$.

Atom	x (Å)	y (Å)	z (Å)	Partial Net Charge (e)
Ni	11.480625	10.697512	7.367602	0.010266
Ni	9.136417	10.069448	7.586347	0.032002
Ni	10.908180	8.437342	8.024422	0.030614
Ni	9.891607	12.258405	8.473792	−0.029576
Ni	12.369668	12.092805	9.049657	−0.046122
Ni	12.753900	9.665505	9.158062	−0.034509
Ni	10.510582	10.238197	9.424103	0.044148
Ni	8.112555	10.933672	9.560992	−0.049843
Ni	8.983530	8.521875	9.549022	−0.034197
Ni	10.914367	14.020987	9.669690	0.009961
Ni	11.248762	7.867372	10.295775	−0.035287
Ni	8.889210	13.034250	10.575405	−0.025443
Ni	10.946655	12.033338	10.897987	0.040857
Ni	13.413375	11.118060	11.069370	−0.037188
Ni	7.353877	9.208777	11.112142	0.054478
Ni	9.252270	10.380510	11.556630	−0.044017
Ni	11.555235	9.789907	11.509560	0.009113
Ni	7.358625	11.635402	11.726212	0.045214
Ni	9.429210	8.151097	11.894895	−0.010136
Ni	10.649970	14.021167	12.059212	0.007612
Ni	9.447412	12.214192	12.917182	−0.024927
Ni	11.892892	12.079372	12.994672	−0.040899
Ni	13.020457	10.010295	13.259925	0.004551
Ni	10.597860	10.118835	13.567410	0.031390
Pd	13.012807	13.581180	11.094435	0.058780
Pd	13.726147	8.575447	11.196292	0.032160
Pd	11.669647	7.770825	12.935002	0.000996

Table S-25: Atomic coordinates and respective DDEC6 partial net charges of each atom in Ni_{27}.

Atom	*x* (Å)	*y* (Å)	*z* (Å)	Partial Net Charge (e)
Ni	11.384707	10.577092	7.249230	0.011833
Ni	9.036697	9.951075	7.491105	0.027224
Ni	10.803150	8.310712	7.922722	0.029037
Ni	9.778702	12.140932	8.359290	−0.031616
Ni	12.264660	11.952202	8.958105	−0.043102
Ni	12.669143	9.532777	9.081832	−0.032669
Ni	10.429312	10.126980	9.305842	0.038159
Ni	7.995352	10.828125	9.445140	−0.046603
Ni	8.884890	8.423932	9.458303	−0.045919
Ni	10.818337	13.913415	9.536693	0.023542
Ni	11.168662	7.792515	10.205393	−0.025112
Ni	8.755110	12.931335	10.456177	−0.020598
Ni	10.796602	11.941965	10.766430	0.040608
Ni	13.353345	10.991160	10.963710	−0.019213
Ni	12.694905	13.269330	10.961550	0.021474
Ni	7.244707	9.085905	11.000047	0.057969
Ni	13.387477	8.536410	11.120647	0.020444
Ni	9.138892	10.269607	11.429122	−0.052613
Ni	11.458687	9.757372	11.405205	0.019968
Ni	7.260615	11.533252	11.632635	0.045612
Ni	9.388080	8.045640	11.810002	−0.002703
Ni	10.578397	13.897777	11.960190	0.014060
Ni	11.593867	7.836030	12.642817	−0.009447
Ni	9.353858	12.097507	12.813255	−0.028179
Ni	11.809125	11.911162	12.850043	−0.037575
Ni	12.927195	9.824692	13.149742	0.009889
Ni	10.453140	9.948712	13.452435	0.035532

S-5 Tables With Cartesian Coordinates and Net Charge Distribution of Adsorbed Configurations

S-5.1 13-atoms Unary and Binary NiPd Clusters

Table S-26: Atomic coordinates and respective DDEC6 partial net charges of each atom in $Pd_{13}H$.

Atom	x (Å)	y (Å)	z (Å)	Partial Net Charge (e)
H	11.532507	12.545505	9.537969	−0.112586
Pd	10.895346	11.591265	11.007906	0.033439
Pd	9.986592	12.618921	8.691858	0.089553
Pd	10.005030	9.969435	8.784867	−0.029364
Pd	10.180191	7.427028	9.498048	0.016901
Pd	12.354048	11.121537	8.804166	0.053843
Pd	12.080103	9.081429	10.603530	−0.029267
Pd	8.269590	11.383596	10.291302	−0.031677
Pd	7.770336	8.786379	9.554118	−0.001635
Pd	9.069459	11.245668	12.901644	0.008925
Pd	7.031346	9.776928	11.958177	0.017517
Pd	11.439561	9.977667	12.982872	0.008703
Pd	9.518355	9.030252	11.482422	−0.019429
Pd	12.126471	8.527092	8.047641	−0.004923

Table S-27: Atomic coordinates and respective DDEC6 partial net charges of each atom in $NiPd_{12}H$.

Atom	x (Å)	y (Å)	z (Å)	Partial Net Charge (e)
H	12.869073	9.767583	12.162717	−0.132291
Ni	10.885098	11.852568	10.845345	−0.074359
Pd	10.147431	13.158600	8.867922	0.015313
Pd	10.051482	10.522239	8.744337	−0.015566
Pd	10.284939	7.983465	9.447375	−0.010009
Pd	12.428493	11.656218	8.901774	0.050996
Pd	12.274794	9.531144	10.564911	0.049613
Pd	8.263290	11.923716	10.179141	−0.020040
Pd	7.851102	9.296994	9.565185	0.013633
Pd	9.176517	11.942049	12.707835	0.037030
Pd	7.174461	10.332924	11.963637	0.007182
Pd	11.578287	10.602732	12.883458	0.091194
Pd	9.669324	9.599310	11.430300	−0.015957
Pd	12.207930	9.178827	7.979328	0.003261

Table S-28: Atomic coordinates and respective DDEC6 partial net charges of each atom in $Ni_3Pd_{10}H$.

Atom	*x* (Å)	*y* (Å)	*z* (Å)	Partial Net Charge (e)
H	7.037856	9.758049	10.421418	−0.141311
Ni	11.768295	9.835413	10.540299	−0.078114
Ni	9.630495	11.080965	10.587423	−0.065361
Ni	11.627868	12.054693	11.277105	−0.031025
Pd	10.534272	12.919704	9.218160	0.040095
Pd	10.495359	10.258311	8.438598	−0.011873
Pd	10.743495	7.727769	9.737028	−0.015300
Pd	12.829173	11.602773	9.144702	0.043010
Pd	8.434419	9.061878	9.732912	0.075253
Pd	9.915276	11.645445	13.026258	0.019193
Pd	7.598934	10.493637	11.872644	0.095230
Pd	12.180021	10.247139	12.938919	0.057339
Pd	9.944382	9.068346	12.012441	−0.015274
Pd	12.799353	8.842302	8.543430	0.028139

Table S-29: Atomic coordinates and respective DDEC6 partial net charges of each atom in Ni_5Pd_8H.

Atom	*x* (Å)	*y* (Å)	*z* (Å)	Partial Net Charge (e)
H	7.959735	8.062572	9.524529	−0.125413
Ni	10.996398	9.156357	9.345756	−0.039660
Ni	10.021221	9.729195	11.420136	−0.034838
Ni	12.360033	10.333617	10.992954	0.009618
Ni	11.776863	11.464698	9.001335	−0.006213
Ni	10.760274	12.066033	11.126871	−0.008353
Pd	9.238110	11.227146	9.256086	0.003451
Pd	10.632069	10.433514	7.179207	−0.000699
Pd	8.474109	11.717664	11.716173	−0.000808
Pd	11.883417	8.037162	11.407242	0.001129
Pd	9.473583	7.460628	10.434795	0.072901
Pd	7.630581	9.480639	10.575474	0.047427
Pd	8.790159	8.792700	8.139054	0.049624
Pd	13.068447	12.630744	10.679382	0.031836

Table S-30: Atomic coordinates and respective DDEC6 partial net charges of each atom in Ni_7Pd_6H.

Atom	x (Å)	y (Å)	z (Å)	Partial Net Charge (e)
H	13.541430	10.848999	8.742300	−0.168656
Ni	10.377024	10.726527	9.504096	−0.059559
Ni	12.412575	11.871447	9.443532	0.113406
Ni	12.117609	10.100055	11.064627	0.004601
Ni	8.811474	12.005805	10.806915	−0.016663
Ni	8.335215	9.738960	10.266480	0.052037
Ni	11.071200	12.221349	11.461359	−0.059427
Ni	9.498783	11.841207	13.211982	0.040702
Pd	10.333701	13.257006	9.343467	0.035536
Pd	10.321773	8.270787	9.890076	−0.039148
Pd	7.342986	10.747653	12.306966	−0.018976
Pd	11.885034	10.949379	13.395018	0.024500
Pd	9.877896	9.691458	12.133863	−0.021852
Pd	12.462408	9.496179	8.744400	0.113498

Table S-31: Atomic coordinates and respective DDEC6 partial net charges of each atom in Ni_8Pd_5H.

Atom	x (Å)	y (Å)	z (Å)	Partial Net Charge (e)
H	7.984305	8.017926	9.516192	−0.142698
Ni	9.366441	11.086950	9.467787	−0.052389
Ni	8.502438	11.740659	11.531373	0.037905
Ni	9.975063	9.936948	11.431119	−0.052091
Ni	9.471126	7.854315	10.371228	0.073184
Ni	8.858829	9.004590	8.411361	0.072884
Ni	12.307050	10.448592	11.032077	0.003570
Ni	11.687571	11.619972	9.031848	0.004122
Ni	10.693746	12.207552	11.124498	−0.003455
Pd	10.324314	10.607898	7.209573	0.005498
Pd	11.312406	9.079245	9.100224	−0.019833
Pd	11.661762	8.091111	11.506257	0.005261
Pd	7.623000	9.606660	10.556868	0.081533
Pd	13.021512	12.747462	10.718946	−0.013490

Table S-32: Atomic coordinates and respective DDEC6 partial net charges of each atom in $Ni_{10}Pd_3H$.

Atom	*x* (Å)	*y* (Å)	*z* (Å)	Partial Net Charge (e)
H	12.453063	9.363816	11.813508	−0.105850
Ni	9.987831	12.524631	8.686965	0.084695
Ni	9.934806	10.194807	8.679153	−0.099747
Ni	10.086405	7.998375	9.370179	0.035644
Ni	11.981067	11.252955	8.739024	0.039301
Ni	11.856831	9.388197	10.315725	−0.002838
Ni	8.416485	11.413500	10.020570	−0.034786
Ni	8.014755	9.087603	9.405228	0.032874
Ni	10.730370	11.550693	10.649688	−0.065558
Ni	11.245605	10.191027	12.439917	0.111017
Ni	9.546096	9.446703	11.107488	−0.047126
Pd	9.142581	11.515392	12.590697	0.002389
Pd	7.108017	9.969477	11.528790	0.019398
Pd	11.911410	8.875839	7.886151	0.030587

Table S-33: Atomic coordinates and respective DDEC6 partial net charges of each atom in $Ni_{12}PdH$.

Atom	*x* (Å)	*y* (Å)	*z* (Å)	Partial Net Charge (e)
H	7.784007	7.958034	9.269946	−0.125294
Ni	9.210684	10.961160	9.095730	−0.059399
Ni	10.249449	10.331391	7.125783	0.033993
Ni	10.835958	9.158289	9.047157	−0.047358
Ni	11.452560	8.056776	11.000892	0.033188
Ni	9.862713	9.726276	11.200665	−0.060053
Ni	9.312912	7.693413	10.090731	0.076882
Ni	7.714329	9.421692	10.168578	0.057003
Ni	8.695974	8.860488	8.102031	0.075568
Ni	12.157845	10.260432	10.720458	−0.011765
Ni	11.539920	11.429628	8.727012	−0.011842
Ni	10.555608	12.030249	10.819473	−0.014519
Ni	12.759369	12.439035	10.376016	0.041906
Pd	8.183931	11.576586	11.294829	0.011688

Table S-34: Atomic coordinates and respective DDEC6 partial net charges of each atom in $Ni_{13}H$.

Atom	x (Å)	y (Å)	z (Å)	Partial Net Charge (e)
H	7.792155	7.945098	9.217005	−0.128065
Ni	9.192330	10.944444	9.058770	−0.056589
Ni	10.235778	10.306863	7.067739	0.034982
Ni	8.308965	11.459280	11.142726	0.033186
Ni	10.896018	9.089157	8.950452	−0.055378
Ni	11.439876	8.036448	10.948623	0.033019
Ni	9.842469	9.718191	11.061645	−0.044732
Ni	7.694463	9.428538	10.129140	0.069410
Ni	8.705760	8.849274	8.062488	0.062785
Ni	12.156795	10.237962	10.656156	−0.018985
Ni	11.523750	11.395587	8.661912	−0.019686
Ni	10.545507	11.998413	10.756242	−0.018808
Ni	12.750360	12.419820	10.305981	0.040082
Ni	9.306927	7.666029	10.027458	0.068781

S-5.2 27-atoms Unary and Binary NiPd Clusters

Table S-35: Atomic coordinates and respective DDEC6 partial net charges of each atom in $Pd_{27}H$.

Atom	x (Å)	y (Å)	z (Å)	Partial Net Charge (e)
H	12.653730	10.340842	14.615842	−0.116084
Pd	7.637760	11.300220	13.072275	0.033040
Pd	8.114332	9.319860	11.197822	−0.023269
Pd	8.488372	11.954610	10.719135	−0.038643
Pd	8.557762	10.378777	8.578282	−0.032097
Pd	8.606160	8.976690	13.874985	−0.017958
Pd	9.056137	13.104135	8.501850	0.041630
Pd	9.526410	11.220007	14.923170	−0.011630
Pd	9.650813	13.058640	12.965422	−0.037176
Pd	10.031512	11.610967	6.594457	−0.008905
Pd	10.153710	7.719097	12.184357	0.031456
Pd	10.302165	10.632690	12.108375	0.109164
Pd	10.275502	8.669835	9.796387	−0.038402
Pd	10.648530	13.532332	10.544152	−0.049333
Pd	10.672177	9.148635	7.284532	0.030259
Pd	11.017890	11.153497	9.360247	0.115213
Pd	11.306632	9.250380	14.121810	0.035293
Pd	11.707335	13.255267	7.914510	−0.023310
Pd	11.908372	11.911725	13.952992	0.014008
Pd	12.094020	14.207377	12.625290	0.028768
Pd	12.325297	9.012802	11.483550	−0.051535
Pd	12.578535	10.929015	6.768180	−0.007005
Pd	12.820117	11.868052	11.492730	−0.020945
Pd	12.810960	9.237262	8.861152	−0.030639
Pd	13.126815	14.179252	10.035090	0.017803
Pd	13.652572	11.794927	9.023962	−0.038309
Pd	13.842247	10.124460	13.315792	0.064075
Pd	14.653417	10.012500	10.725525	0.024532

Table S-36: Atomic coordinates and respective DDEC6 partial net charges of each atom in $Ni_3Pd_{24}H$.

Atom	x (Å)	y (Å)	z (Å)	Partial Net Charge (e)
H	10.770322	11.766352	14.312880	−0.115535
Ni	10.448797	10.614037	9.483232	0.006177
Ni	9.717030	8.447625	9.392130	−0.083005
Ni	10.675845	12.268440	11.185087	−0.022787
Pd	11.690325	10.830510	7.122802	0.020816
Pd	9.147600	9.955732	7.479720	0.031682
Pd	11.398725	8.173372	7.644870	0.024328
Pd	9.959445	12.649095	8.084070	−0.027875
Pd	12.381187	12.323362	9.290722	0.019851
Pd	12.903255	9.685395	9.192060	−0.020270
Pd	8.039542	11.305237	9.516465	−0.020492
Pd	11.258977	14.730052	9.454567	0.007151
Pd	11.810925	7.422232	10.265850	−0.030561
Pd	8.981775	13.772632	10.311435	−0.029045
Pd	13.875052	11.224642	11.227117	−0.042094
Pd	13.303170	13.860652	11.202817	0.002798
Pd	7.558470	8.839777	10.414372	0.054274
Pd	14.044657	8.553375	11.327760	0.008900
Pd	8.908245	10.493392	11.909115	−0.010861
Pd	11.600932	9.885780	11.515995	0.138061
Pd	7.316325	12.539497	11.819880	0.001740
Pd	9.695092	8.011507	11.889157	−0.013028
Pd	10.860907	14.502420	12.125250	−0.000889
Pd	12.060967	7.654500	12.998745	−0.026038
Pd	9.475223	12.580200	13.411912	0.041105
Pd	12.186855	12.235882	13.153410	0.038054
Pd	13.445640	9.915187	13.575577	−0.004586
Pd	10.681177	9.999652	13.941225	0.052130

Table S-37: Atomic coordinates and respective DDEC6 partial net charges of each atom in $Ni_7Pd_{20}H$.

Atom	x (Å)	y (Å)	z (Å)	Partial Net Charge (e)
H	10.925662	13.552807	13.666657	−0.128637
Ni	12.849075	13.465980	11.085030	−0.014571
Ni	13.314712	11.098867	11.179012	−0.066891
Ni	9.296415	8.549167	9.553410	−0.066065
Ni	10.526085	10.477012	9.369157	0.022105
Ni	11.032222	12.011557	11.080327	0.022499
Ni	9.233572	10.435950	11.623207	−0.075475
Ni	11.484045	9.600480	11.373142	0.014419
Pd	10.986637	8.141152	7.779487	0.017861
Pd	12.834022	9.570465	9.054990	−0.006213
Pd	9.565222	7.984732	12.001972	0.018877
Pd	13.870012	8.589937	11.221582	0.037363
Pd	11.596162	10.683090	6.972277	0.006415
Pd	7.307280	9.026662	11.022952	0.046139
Pd	7.288267	11.918992	11.886120	0.013373
Pd	8.068905	11.080057	9.502650	0.022124
Pd	12.264615	12.372750	13.294710	0.057938
Pd	11.982442	7.650832	12.980047	−0.041282
Pd	9.895387	12.518730	8.080065	−0.050607
Pd	10.654695	10.023255	13.663260	0.033160
Pd	9.085500	13.366867	10.555942	−0.036356
Pd	10.942065	14.577322	12.175268	0.068605
Pd	11.199960	14.483925	9.447390	−0.002401
Pd	9.015480	9.938947	7.358152	0.030560
Pd	9.551070	12.445020	13.173075	0.044767
Pd	12.506512	12.199500	9.003802	0.039006
Pd	11.534985	7.391025	10.261777	−0.023909
Pd	13.333612	9.976162	13.433130	0.017195

Table S-38: Atomic coordinates and respective DDEC6 partial net charges of each atom in $Ni_{10}Pd_{17}H$.

Atom	x (Å)	y (Å)	z (Å)	Partial Net Charge (e)
H	10.406497	8.240062	13.590810	−0.140324
Ni	13.183447	10.101870	13.193662	−0.049118
Ni	9.765810	12.421395	12.970372	−0.081117
Ni	11.549452	7.914240	10.324395	−0.052385
Ni	11.438077	10.724107	7.342042	−0.058097
Ni	9.323077	13.170397	10.504485	−0.058350
Ni	8.287335	11.232382	9.483930	−0.066967
Ni	11.304135	12.218445	11.148165	0.024832
Ni	10.599952	10.624207	9.455108	0.035947
Ni	9.377010	10.635120	11.554110	−0.057682
Ni	11.670997	9.887805	11.455762	−0.000062
Pd	13.962330	8.629920	11.258617	−0.014289
Pd	9.264487	8.579610	9.384187	−0.015983
Pd	12.443782	12.418897	8.935560	0.008032
Pd	10.643220	10.133640	13.730445	0.129179
Pd	12.958605	9.786870	9.113580	0.014433
Pd	11.257830	8.239185	7.743487	0.047350
Pd	10.997347	14.612805	9.442642	−0.006323
Pd	13.097790	13.957425	11.053057	−0.029172
Pd	7.478820	12.189667	11.803185	0.110536
Pd	9.826897	12.640455	8.033377	0.023058
Pd	7.295692	9.442440	10.934640	0.013389
Pd	12.002377	7.801852	12.912862	0.074005
Pd	9.008235	10.185142	7.276230	0.040500
Pd	13.768717	11.388487	11.164297	0.008560
Pd	12.235792	12.426210	13.436145	0.003887
Pd	9.499792	8.220330	12.027375	0.049050
Pd	10.685430	14.532840	12.169485	0.047112

Table S-39: Atomic coordinates and respective DDEC6 partial net charges of each atom in $Ni_{14}Pd_{13}H$.

Atom	x (Å)	y (Å)	z (Å)	Partial Net Charge (e)
H	9.678240	13.803817	9.089235	−0.083224
Ni	9.644918	12.215565	13.092412	−0.046888
Ni	12.814110	9.645795	9.365243	−0.038273
Ni	7.463610	9.109372	11.360812	0.026251
Ni	11.203515	13.952002	9.805883	0.037092
Ni	10.173645	12.173130	8.628705	−0.020754
Ni	13.511025	11.040772	11.299590	−0.049537
Ni	11.089012	8.452260	8.114782	−0.004208
Ni	12.577027	12.042855	9.324540	−0.056041
Ni	9.387472	10.070145	7.654005	−0.014558
Ni	10.790235	10.068750	13.706527	0.025750
Ni	11.071935	11.970067	11.105010	0.042312
Ni	9.361350	10.341788	11.830207	−0.065144
Ni	11.682967	9.667867	11.643232	0.002366
Ni	10.513080	10.095345	9.657630	0.035727
Pd	13.936500	8.556457	11.286877	0.029976
Pd	7.324425	11.689627	12.156457	0.026240
Pd	11.826585	10.761030	7.313040	0.087584
Pd	8.895487	13.207005	10.628550	0.043116
Pd	13.255807	13.528867	11.199802	0.039934
Pd	9.525712	7.971390	12.169710	0.014992
Pd	11.945407	7.720807	13.171995	−0.020127
Pd	8.963122	8.181495	9.503820	−0.018173
Pd	13.385092	9.961807	13.619745	0.015629
Pd	10.822612	14.301810	12.300930	0.008562
Pd	12.120615	12.233992	13.368825	−0.008229
Pd	11.531880	7.524202	10.364737	−0.018706
Pd	8.073855	10.835910	9.624015	0.008332

Table S-40: Atomic coordinates and respective DDEC6 partial net charges of each atom in $Ni_{17}Pd_{10}H$.

Atom	x (Å)	y (Å)	z (Å)	Partial Net Charge (e)
H	8.475615	8.063978	11.105257	−0.079864
Ni	11.621452	8.218192	10.464975	−0.039009
Ni	8.345992	11.238412	9.739507	−0.067978
Ni	12.980407	9.993015	9.370057	−0.051845
Ni	10.964632	14.211067	12.234825	−0.019060
Ni	9.251640	8.663355	9.668475	0.026594
Ni	9.847260	8.650642	12.013987	0.020512
Ni	12.006270	8.194860	12.826080	−0.001053
Ni	11.713455	10.980990	7.554645	−0.022635
Ni	7.767832	9.560092	11.309242	0.080712
Ni	10.209645	12.521700	8.595405	−0.039097
Ni	13.752045	8.901180	11.361735	0.028968
Ni	13.069845	13.790227	11.305777	0.015936
Ni	12.080633	12.277710	13.021695	−0.053571
Ni	11.841637	10.217835	11.674732	−0.006913
Ni	11.301345	12.388725	10.808932	0.024019
Ni	10.691190	10.447357	9.608783	0.025660
Ni	9.562387	10.973227	11.757487	−0.046205
Pd	9.553477	12.685027	13.525357	0.012551
Pd	13.975785	11.466495	11.227792	0.000970
Pd	11.264287	8.519760	8.024850	0.041387
Pd	9.063832	13.482765	10.701652	0.019576
Pd	7.249590	11.911860	11.939265	0.010581
Pd	13.458105	10.192163	13.601745	0.016226
Pd	10.668982	10.181025	13.919152	0.028601
Pd	12.973680	12.564900	8.966880	0.007499
Pd	11.270002	14.644980	9.641835	0.018947
Pd	9.205942	10.344937	7.589992	0.048489

Table S-41: Atomic coordinates and respective DDEC6 partial net charges of each atom in $Ni_{20}Pd_7H$.

Atom	x (Å)	y (Å)	z (Å)	Partial Net Charge (e)
H	13.085730	11.055060	8.250975	−0.070783
Ni	11.522160	10.849005	7.624980	0.061838
Ni	9.223110	10.243822	7.725937	−0.002169
Ni	10.943730	8.591692	8.166600	−0.009152
Ni	9.951728	12.350317	8.712180	−0.057211
Ni	12.442027	12.260385	9.315562	0.000629
Ni	12.777637	9.815355	9.417262	0.025847
Ni	10.485202	10.304055	9.662895	0.027741
Ni	8.934457	13.182975	10.866262	−0.046707
Ni	10.974735	12.121627	11.118195	0.033932
Ni	13.369432	11.253195	11.376720	−0.036211
Ni	12.841852	13.528350	11.361285	0.021386
Ni	9.251865	10.553017	11.754315	−0.046962
Ni	11.525287	9.848475	11.744055	−0.003481
Ni	7.396942	11.876827	12.118815	0.041362
Ni	9.555120	8.353035	12.217072	−0.047081
Ni	10.711417	14.116657	12.327705	0.015088
Ni	9.490455	12.341002	13.168170	−0.014860
Ni	11.905515	12.187372	13.224352	−0.039724
Ni	12.999398	10.153777	13.513747	0.009207
Ni	10.538595	10.217767	13.770900	0.050219
Pd	7.933522	11.122155	9.706702	0.017351
Pd	8.855955	8.437995	9.644130	−0.010596
Pd	10.985490	14.357767	9.787320	0.054169
Pd	11.401402	7.750732	10.449405	−0.008213
Pd	7.216830	9.217552	11.650612	0.014242
Pd	13.776885	8.758552	11.508367	0.008236
Pd	11.744415	7.916670	13.300605	0.011899

Table S-42: Atomic coordinates and respective DDEC6 partial net charges of each atom in $Ni_{24}Pd_3H$.

Atom	x (Å)	y (Å)	z (Å)	Partial Net Charge (e)
H	12.892927	10.899382	8.081482	−0.071579
Ni	11.331225	10.683675	7.495402	0.049548
Ni	9.022770	10.034145	7.716060	0.024958
Ni	10.788660	8.412457	8.141175	0.026809
Ni	9.775732	12.214935	8.610277	−0.041344
Ni	12.283312	12.080723	9.187740	−0.009220
Ni	12.653010	9.645952	9.260122	0.012534
Ni	10.392232	10.194817	9.569272	0.047894
Ni	8.023477	10.904377	9.694912	−0.053095
Ni	8.882842	8.467785	9.641722	−0.034303
Ni	10.816740	13.970070	9.768150	0.007751
Ni	11.153385	7.842622	10.383412	−0.047203
Ni	8.797770	13.006620	10.688602	−0.027783
Ni	10.839825	11.988742	11.030760	0.036095
Ni	13.284855	11.074027	11.230380	−0.036582
Ni	7.263000	9.124020	11.214382	0.060715
Ni	9.129757	10.332720	11.707200	−0.050925
Ni	11.421382	9.753502	11.640375	0.002777
Ni	7.255012	11.630025	11.843977	0.057725
Ni	9.339547	8.095230	11.974680	−0.012909
Ni	10.563615	14.004945	12.157852	0.010596
Ni	9.368707	12.214350	13.011682	−0.026276
Ni	11.803252	12.074288	13.119120	−0.041038
Ni	12.892972	9.992025	13.393957	0.004365
Ni	10.494945	10.141357	13.694872	0.032259
Pd	12.902602	13.540995	11.211727	0.051757
Pd	13.575735	8.548717	11.313922	0.020092
Pd	11.540520	7.745152	13.053915	0.006383

Table S-43: Atomic coordinates and respective DDEC6 partial net charges of each atom in $Ni_{27}H$.

Atom	x (Å)	y (Å)	z (Å)	Partial Net Charge (e)
H	12.765307	10.689367	7.950892	−0.078861
Ni	11.200927	10.497172	7.366387	0.049111
Ni	8.883225	9.901035	7.630447	0.024589
Ni	10.622722	8.251470	8.061165	0.020035
Ni	9.679973	12.078765	8.478900	−0.042704
Ni	12.175942	11.906190	9.052312	0.001186
Ni	12.522240	9.470587	9.174330	0.019146
Ni	10.270665	10.058355	9.463928	0.044488
Ni	7.918717	10.822005	9.604373	−0.049930
Ni	8.752050	8.352720	9.595913	−0.036023
Ni	10.757317	13.851315	9.594180	0.011728
Ni	11.064172	7.750125	10.321132	−0.035923
Ni	8.694270	12.947242	10.545502	−0.023439
Ni	10.694565	11.890890	10.868715	0.035402
Ni	13.178047	10.954215	11.081767	−0.025652
Ni	12.623603	13.252860	11.011320	0.024016
Ni	7.150590	9.085882	11.147220	0.054719
Ni	13.234455	8.522167	11.235397	0.008179
Ni	9.002272	10.282545	11.619720	−0.060715
Ni	11.295540	9.704497	11.549160	0.008422
Ni	7.156192	11.591280	11.742052	0.055658
Ni	9.288810	8.062605	11.939130	−0.007918
Ni	10.536390	13.909477	11.995132	0.019661
Ni	11.476372	7.812832	12.786547	−0.001208
Ni	9.308160	12.156885	12.906630	−0.027855
Ni	11.741445	11.930872	12.929760	−0.037570
Ni	12.808957	9.816818	13.250430	0.007126
Ni	10.371037	10.007977	13.604917	0.044330

References

1 Kresse, G.; Furthmüller, J. Efficient Iterative Schemes for *Ab initio* Total-energy Calculations Using a Plane-wave Basis set. *Phys. Rev. B* **1996**, *54*, 11169–11186, DOI: 10.1103/physrevb.54.11169.

2 Hohenberg, P.; Kohn, W. Inhomogeneous Electron Gas. *Phys. Rev.* **1964**, *136*, B864–B871, DOI: 10.1103/physrev.136.b864.

3 Kohn, W.; Sham, L. J. Self-Consistent Equations Including Exchange and Correlation Effects. *Phys. Rev.* **1965**, *140*, A1133–A1138, DOI: 10.1103/physrev.140.a1133.

4 Perdew, J. P.; Burke, K.; Ernzerhof, M. Generalized Gradient Approximation Made Simple. *Phys. Rev. Lett.* **1996**, *77*, 3865–3868, DOI: 10.1103/physrevlett.77.3865.

5 Blöchl, P. E. Projector Augmented-Wave Method. *Phys. Rev. B* **1994**, *50*, 17953–17979, DOI: 10.1103/physrevb.50.17953.

6 Kresse, G.; Joubert, D. From Ultrasoft Pseudopotentials to the Projector Augmented-wave Method. *Phys. Rev. B* **1999**, *59*, 1758–1775, DOI: 10.1103/physrevb.59.1758.

7 Grimme, S.; Antony, J.; Ehrlich, S.; Krieg, H. A Consistent and Accurate ab Initio Parametrization of Density Functional Dispersion Correction (DFT-D) for the 94 Elements H–Pu. *J. Chem. Phys.* **2010**, *132*, 154104, DOI: 10.1063/1.3382344.

8 Marques, J. M. C.; Pereira, F. B. An evolutionary algorithm for global minimum search of binary atomic clusters. *Chem. Phys. Lett.* **2010**, *485*, 211.

9 Da Silva, J. L. F. Effective Coordination Concept Applied for Phase Change $(GeTe)_m(Sb_2Te_3)_n$ Compounds. *J. Appl. Phys.* **2011**, *109*, 023502, DOI: 10.1063/1.3533422.

10 Chaves, A. S.; Rondina, G. G.; Piotrowski, M. J.; Da Silva, J. L. F. Structural Formation of Binary PtCu Clusters: A Density Functional Theory Investigation. *Compt. Mater. Sci.* **2015**, *98*, 278–286, DOI: 10.1016/j.commatsci.2014.11.022.

11 da Silva, L. R.; Morais, F. O.; de Mendonça, J. P. A.; Galvão, B. R. L.; Da Silva, J. L. F. Theoretical investigation of the stability of $A_{55-n}B_n$ nanoalloys (A,B= Al, Cu, Zn, Ag). *Comput. Mater. Sci.* **2022**, *215*, 111805, DOI: 10.1016/j.commatsci.2022.111805.

12 Ocampo-Restrepo, V. K.; Verga, L. G.; Da Silva, J. L. F. Ab initio Study of the C–O Bond Dissociation in CO_2 Reduction by Redox and Carboxyl Routes on 3d Transition Metal Systems. *J. Phys. Chem. C* **2021**, *125*, 26296–26306, DOI: 10.1021/acs.jpcc.1c05468.

13 Fournier, R.; Salahub, D. Chemisorption and magnetization: a bond order-rigid band model. *Surf. Sci.* **1990**, *238*, 330–340, DOI: 10.1016/0039-6028(90)90091-L.

14 Mao, X.; Wang, L.; Xu, Y.; Wang, P.; Li, Y.; Zhao, J. Computational high-throughput screening of alloy nanoclusters for electrocatalytic hydrogen evolution. *npj Comput. Mater.* **2021**, *7*, 46, DOI: 10.1038/s41524-021-00514-8.

15 Reimer, W.; Penka, V.; Skottke, M.; Behm, R. J.; Ertl, G.; Moritz, W. A leed analysis of the (2× 1) H-Ni(110) structure. *Surf. Sci.* **1987**, *186*, 45–54, DOI: 10.1016/S0039-6028(87)80034-1.

16 Felter, T. E.; Sowa, E. C.; Van Hove, M. A. Location of hydrogen adsorbed on palladium (111) studied by low-energy electron diffraction. *Phys. Rev. B* **1989**, *40*, 891, DOI: 10.1103/PhysRevB.40.891.

17 Greeley, J.; Mavrikakis, M. Surface and Subsurface Hydrogen: Adsorption Properties on Transition Metals and Near-Surface Alloys. *J. Phys. Chem. B* **2005**, *109*, 3460–3471, DOI: 10.1021/jp046540q.

18 Khalid, M.; Fonseca, H. A.; Verga, L. G.; Hatshan, M. R.; Da Silva, J. L. F.; Varela, H.; Shahgaldi, S. Facile synthesis of Ru nanoclusters embedded in carbonaceous shells for hydrogen evolution reaction in alkaline and acidic media. *J. Electroanal. Chem.* **2023**, *929*, 117116, DOI: 10.1016/j.jelechem.2022.117116.

19 Karthick, K.; Bijoy, T.; Sivakumaran, A.; Mansoor Basha, A. B.; Murugan, P.; Kundu, S. Enhancing hydrogen evolution reaction activities of 2H-phase VS_2

layers with palladium nanoparticles. *Inorg. Chem.* **2020**, *59*, 10197–10207, DOI: 10.1021/acs.inorgchem.0c01339.

20 Cui, J.; Liu, X.; Wang, Y.; Song, D.; Ren, Y.; Shen, X. Hydrogen evolution reaction on transition metal nanoparticles from first-principles. *Appl. Surf. Sci.* **2021**, *570*, 151211, DOI: 10.1016/j.apsusc.2021.151211.

21 Dubouis, N.; Grimaud, A. The hydrogen evolution reaction: from material to interfacial descriptors. *Chem. Sci.* **2019**, *10*, 9165–9181, DOI: 10.1039/c9sc03831k.

22 Zhang, B.; Liu, J.; Wang, J.; Ruan, Y.; Ji, X.; Xu, K.; Chen, C.; Wan, H.; Miao, L.; Jiang, J. Interface engineering: the $Ni(OH)_2/MoS_2$ heterostructure for highly efficient alkaline hydrogen evolution. *Nano Energy* **2017**, *37*, 74–80, DOI: 10.1016/j.nanoen.2017.05.011.

23 Lv, X.; Wei, W.; Wang, H.; Huang, B.; Dai, Y. Multifunctional electrocatalyst PtM with low Pt loading and high activity towards hydrogen and oxygen electrode reactions: A computational study. *Appl. Catal., B* **2019**, *255*, 117743, DOI: 10.1016/j.apcatb.2019.05.045.

24 Bornemann, S.; Šipr, O.; Mankovsky, S.; Polesya, S.; Staunton, J.; Wurth, W.; Ebert, H.; Minár, J. Trends in the magnetic properties of Fe, Co, and Ni clusters and monolayers on Ir (111), Pt (111), and Au (111). *Physical Review B* **2012**, *86*, 104436, DOI: 10.1103/PhysRevB.86.104436.

25 Aguilera-Granja, F.; Bouarab, S.; López, M. J.; Vega, A.; Montejano-Carrizales, J. M.; Iñiguez, M. P.; Alonso, J. A. Magnetic moments of Ni clusters. *Phys. Rev. B* **1998**, *57*, 12469.

26 Rogan, J.; García, G.; Ramírez, M.; Munoz, V.; Valdivia, J. A.; Andrade, X.; Ramírez, R.; Kiwi, M. The structure and properties of small Pd clusters. *Nanotechnology* **2008**, *19*, 205701, DOI: 10.1088/0957-4484/19/20/205701.

27 Souza, T. M.; Pena, L. B.; Da Silva, J. L. F.; Galvão, B. R. L. Data-driven stabilization of Ni_mPd_{n-m} nanoalloys: a study using density functional theory and data mining approaches. *Phys. Chem. Chem. Phys.* **2024**, *26*, 15877–15890, DOI: 10.1039/D4CP00672K.

28 Zeng, Z.; Chen, X.; Weng, K.; Wu, Y.; Zhang, P.; Jiang, J.; Li, N. Computational screening study of double transition metal carbonitrides M'2M"CNO_2-MXene as catalysts for hydrogen evolution reaction. *npj Comput. Mater.* **2021**, *7*, 80, DOI: 10.1038/s41524-021-00550-4.
